# The Supertree Toolkit 2: a new and improved software package with a Graphical User Interface for supertree construction

**DOI:** 10.3897/BDJ.2.e1053

**Published:** 2014-03-26

**Authors:** Jon Hill, Katie E Davis

**Affiliations:** † Imperial College London, London, United Kingdom; ‡ University of Bath, Bath, United Kingdom

**Keywords:** Supertree, phylogeny, data curation, meta-data

## Abstract

Building large supertrees involves the collection, storage, and processing of thousands of individual phylogenies to create large phylogenies with thousands to tens of thousands of taxa. Such large phylogenies are useful for macroevolutionary studies, comparative biology and in conservation and biodiversity. No easy to use and fully integrated software package currently exists to carry out this task. Here, we present a new Python-based software package that uses well defined XML schema to manage both data and metadata. It builds on previous versions by 1) including new processing steps, such as Safe Taxonomic Reduction, 2) using a user-friendly GUI that guides the user to complete at least the minimum information required and includes context-sensitive documentation, and 3) a revised storage format that integrates both tree- and meta-data into a single file. These data can then be manipulated according to a well-defined, but flexible, processing pipeline using either the GUI or a command-line based tool. Processing steps include standardising names, deleting or replacing taxa, ensuring adequate taxonomic overlap, ensuring data independence, and safe taxonomic reduction. This software has been successfully used to store and process data consisting of over 1000 trees ready for analyses using standard supertree methods. This software makes large supertree creation a much easier task and provides far greater flexibility for further work.

## Introduction

Supertrees are large phylogenies created by amalgamating anywhere from tens to thousands of smaller source phylogenies. A number of algorithms exist for this, the most widely used being Matrix Representation with Parsimony (Ragan 1992). These algorithms have varying extents of software implementation again with MRP being the most commonly implemented. Supertrees have been created for a wide variety of taxonomic groups, including birds (Cornwallis et al. 2010, Davis 2008), dinosaurs (Lloyd et al. 2008), angiosperms (Davies et al. 2004) and marsupials (Cardillo et al. 2004). Large phylogenies are useful for answering questions in macroevolution (e.g. Lloyd et al. 2008), comparative biology (e.g. Edwards et al. 2007), biodiversity (Crozier et al. 2005, Helmus et al. 2007) and conservation (e.g. Isaac et al. 2007). However, large-scale supertrees are not straightforward to construct. Collecting, storing and processing the source trees which are the input to any supertree algorithm is non-trivial as a number of important considerations must be made concerning data quality. These issues with supertree analysis, including data independence, taxonomic overlap, and consistent taxonomy have been discussed elsewhere at length (Bininda-Emonds et al. 2004, Bininda-Emonds et al. 2005, Gatesy 2004). Given the potential large size of datasets and the amount of processing that must be done prior to the supertree analysis being carried out this is not an easy task. There is therefore a need for a comprehensive software package that can carry out this prior processing and can preferably store data in a well defined manner.

Some workers have written and made available scripts that carry out one or more of the required processing steps (e.g. Bininda-Emonds et al. 2004, Bininda-Emonds et al. 2005, Davis and Hill 2010). These scripts have a number of drawbacks such as the lack of a user friendly interface and lack of optimisation resulting in slow, computationally intensive analyses that take many days or even weeks to run for large datasets. Other issues include data format conversion between processing steps, for example converting from Newick-based tree strings to NEXUS format. In addition none of these software scripts include methodologies for collecting metadata – a key part of a robust and rigorous processing pipeline. An attempt to mitigate these issues led to the creation of the Supertree Toolkit (STK) (Davis and Hill 2010); a collection of Perl scripts designed to carry out the prior processing required for supertree construction. This package was the first to use both source trees alongside their metadata to perform the processing. This software, however, was difficult to use (command-line only) and had only a rudimentary GUI for creating metadata. The storage mechanisms chosen required strict naming conventions and therefore was somewhat fragile. Moreover, the metadata was optional for most of the processing pipeline, thereby negating its full value. The original STK (Davis and Hill 2010), whilst a step in the right direction, did not therefore meet the requirements for an easy-to-use, rigorous method to collect both metadata and data.

Here, we present the next version of the Supertree Toolkit that builds on the experience of the first version. We have rewritten all code and designed the software around a user interface that can carry out both data collection and processing. It contains a number of additional features over the original software which are 1) new processing steps, such as Safe Taxonomic Reduction, 2) user-friendly GUI that guides the user to complete at least the minimum information required and includes context-sensitive documentation, and 3) a revised storage format that integrates both tree- and meta-data into a single file. We will first detail the storage mechanism, based on RelaxNG XML, and the user interface features. We then cover the available processing pipeline steps and show some examples of their use.

## Project description

### Title

Supertree Toolkit (STK)

### Design description

The STK consists of three components: a Python module, a Graphical User Interface (GUI), and a Command Line Interface (CLI). The python module contains all processing, importing and exporting functions. These functions deal with the Phyml format (see below) and are available in any Python environment by importing the supertree_toolkit module. The GUI and CLI then import this Python module and hook it to the interface by processing user options. In this way the core functionality can be tested by using standard unit test infrastructure and the interfaces are cleanly separated. A test suite of over 375 tests is included in the source code which benchmark the expected performance of the software.

**User interface**

**Figure 1.**
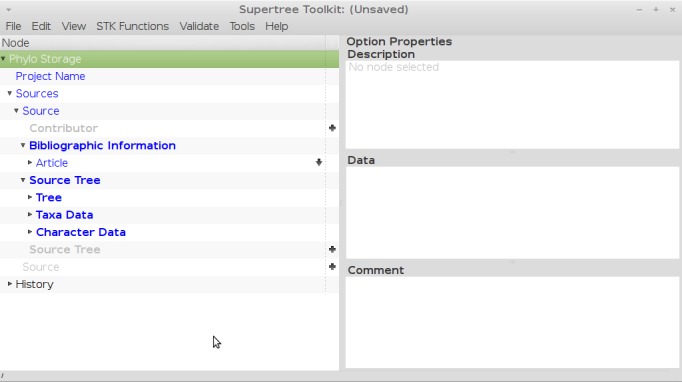
The STK GUI, based on Diamond (Ham et al. 2009). The GUI is split into two main panes, with the left being used to store data and the right showing the context-based help (top), data entry (middle) and comments (bottom).

There are two user interfaces: a GUI for data entry and processing, and a CLI for data processing. The latter is useful for dealing with large datasets. The GUI is based on Diamond (Ham et al. 2009), which was originally designed for entering user options for numerical modelling software. We have extended the capabilities of Diamond to be suitable for entering phylogenetic data. A number of specific plugins have been created to import source trees and manage bibliographic sources. We use the BibTeX format for importing and exporting bibliographic information, which is widely supported by references managers. We have also added tools to carry out data processing based on those in the original STK (Davis and Hill 2010) and extending them to include safe taxonomic reduction (Wilkinson 1995). The GUI is split into two vertical panes; the left contains a hierarchical view of the data. Each row in this hierarchy is an element, which can contain a number of nested elements. Elements at the same level in the hierarchy can be copy and pasted to aid data entry. The right-hand side is split into three horizontal panes (Fig. 1). The uppermost pane displays context-sensitive help, the middle pane allows for data entry and the lower-most pane contains user comments where available. All processing functions are available in the GUI and use specifically designed interfaces to allow the user to choose options and output processing steps to the file. The command line interface contains the processing steps as sub-commands. Each processing step then contains the options specific to this function. As with the GUI, documentation is available using standard flags and options.

We have maintained all the previous functionality of the previous version of the STK which are detailed in Davis and Hill (2010). We therefore restrict ourselves to a brief description of the functionality and describe new functionality in detail (Safe Taxonomic Reduction).

**Metadata and file format**

XML is an ideal way to store structured metadata. We build on the methods used by Spud (Ham et al. 2009), using the RelaxNG method to create a data schema. This schema dictates what information the user interface shows – the options in the left hand side of the GUI (Fig. 1) are generated on the fly from this schema – and aids the user in two ways. First, data can be defined as required or optional within the schema and the interface highlights required data accordingly. Second, context-sensitive documentation is embedded in the schema and is shown in the user interface. The user does not interact directly with the schema, but the schema dictates which data can be stored and what is shown in the user interface. The base schema is a human readable file in compact RelaxNG format. It is this file where required and optional GUI elements can be added as well as context-senstivie documentation. Software (spud-preprocess) distributed as part of Spud (Ham et al. 2009) then transforms this into a full XML file. The two files (one a .rnc the other a .rng) are distributed along with the GUI to generate the user interface. The GUI then reads this XML files (the .rng file otherwise known as the schema) to display options to the user. The file the user saves is therefore linked to this schema as the schema dictates the data that can be displayed and entered via the GUI and hence saved in a file. There are a number of other file formats that allow the user to store both trees and their metadata, such as NexML (Vos et al. 2012) and PhyloXML (Han and Zmasek 2009). We have written the code so that the GUI can be automatically generated from our XML schema, but not NeXML or PhyloXML, however parsers to import from and export to these file formats will be added in future versions. The schema used here is easily extensible and was designed with initiatives such as MIAPA in mind, which was used to define the terms used, though not in a formal manner.

**Figure 2.**
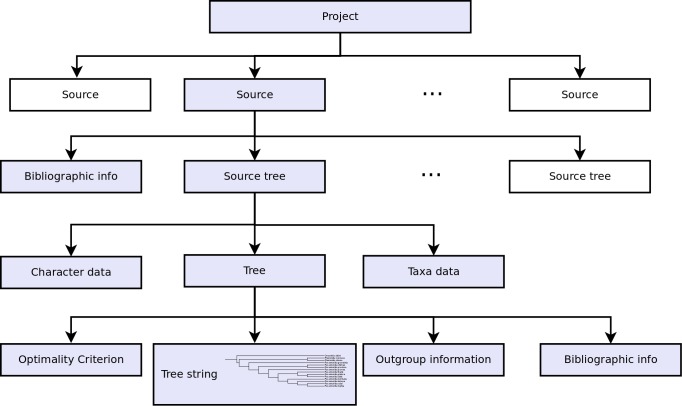
Data structure of the STK metadata. Each project consists of several sources, which in turn contain bibliographic information and one or more source trees. The blue boxes show the hierarchy for a single source tree. The data structure has been simplified here and more meta data can be stored for each source tree.

Each dataset has a name and contains a number of "Sources" (Fig. 2). Each source is a publication and includes the bibliographic information, followed by one or more "Source Trees". Each "Source Tree" then contains the tree string, the characters and methods used to create the tree, along with information on fossil taxa and other metadata (figure number, legend, etc.). Optional information includes conservation status, stratigraphic information, synonyms and database accession numbers. This approach allows copy and pasting of "Sources" between dataset via the GUI, easy navigation of the whole data structure as well as the meta- and source tree data being displayed alongside each other. This is in contrast to the previous version where files were distributed within sub-folders on disk and the tree and meta-data were contained in separate files (Davis and Hill 2010).

The result of this schema is a single XML datafile that contains all metadata and source data required. This file is termed a Phylml (Phylogenetic Meta Language), which can be parsed by any standard XML parser.

**Processing functions**

**Figure 3.**
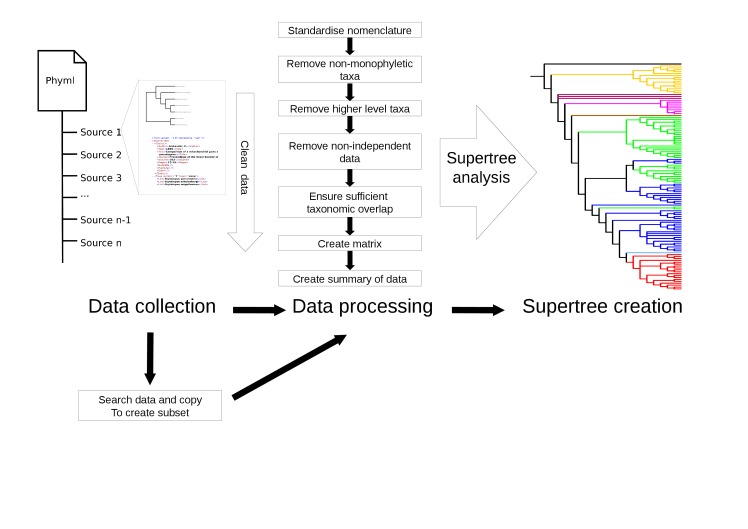
Example of a processing pipeline that can be created with the STK. Data are collected and then are put through the processing pipeline in order to create a matrix. The resulting matrix (in either Nexus format (.nex) or Hennig format (.tnt)) can then be analysed in any suitable software such as PAUP* (Swofford 2003) or TNT (Goloboff et al. 2008).

**Figure 4.**
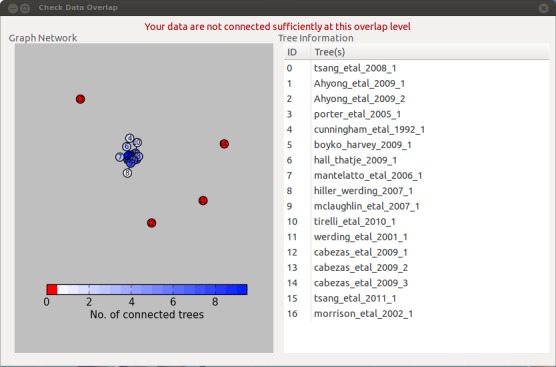
Result of the taxonomic overlap check which highlights which source trees are not sufficiently well connected to the rest of the dataset.

There are a number of processing functions included in the STK. These can be chained together to construct a *processing pipeline* to collect, curate and process data (Fig. 3). The processing functions are:

- Data summary – produce text summary of data, such as number of taxa, trees and characters.
- Clean data – check data and remove redundant data, such as non-informative trees.
- Permute all trees – remove non-monophyly from trees.
- Substitute taxa – perform substitutions or deletions of taxa.
- Data independence check – check that all source trees are independent of each other.
- Data overlap – check the taxonomic overlap (Fig. 4).
- Replace genera – replace generic-level taxa with polytomies of all species in that genus that are in the dataset.
- Create matrix – create a MRP matrix (Baum and Ragan coding) of the dataset.
- Create subset – create a subset of this dataset, e.g. only certain years or data types.
- STR – perform safe taxonomic reduction on the dataset. This is new functionality over the previous version of the STK (Davis and Hill 2010)

**Data summary**

This function creates a text summary of the data. The summary includes a taxa list, years of publication, characters used, and analyses used.

**Clean data**

Before and during processing trees may become uninformative (i.e. contain no clades), for example after substitution of taxa, or when dealing with polyphyletic taxa. This function checks that the data are suitable for processing and removes uninformative trees (and sources if they contain no trees) and should be run regularly on data between processing steps.

**Permute trees**

When creating supertrees at species level digitised trees need to account for the fact that some species may be polyphyletic. There are no formal mechanisms for dealing with this so taxa can be encoded with a '%d' sign to designate them as polyphyletic (where d is a consecutive integer for each taxon). The 'permute trees' function generates all possible permutations of these trees to enable a consensus tree of some kind to be created.

**Substitute taxa**

One of the most onerous tasks of supertree creation is ensuring a consistent taxonomy is used throughout. This requires the removal of synonyms, mis-spellings and other naming errors. The 'sub taxa' function allows substitution and deletion of taxa whilst maintaining the tree structure. Substitutions are aware of polyphyletic taxa and will collapse superfluous nodes when deleting taxa. This function is used throughout the processing.

**Data independence**

It has previously been noted that all data included in supertree analyses should be independent of each other. Here, we defined non-independent data as being datasets that contain a subset of the same taxa and use identical characters. This function flags source trees that are subsets of others (and can automatically remove them if required) and flags those that are identical (i.e. same taxa and characters).

**Replace genera**

This is one of the final steps of the pipeline. After all processing some taxa at genus level may be left in the source trees. This function replaces those genera with a polytomy of species already in the dataset. Note that this assumes a species level tree is to be created and this step can be omitted if this is not the case.

**Data overlap**

In order to create a supertree all source trees should exhibit sufficient taxonomic overlap (Sanderson et al. 1998). Data overlap checks this with a user defined overlap (minimum is two) and can present the results in two graphical formats (e.g. Fig. 4).

**Create subset**

One of the novelties of the STK is that it can be used to create subsets of the whole dataset, based on the metadata. For example, all trees that used molecular character can be extracted and used to create a new dataset. Similarly publication year, author, or analysis type can all be used to create subsets. These can be used to create independent supertrees and the effect of including, say, only molecular data, can be compared to the supertree generated from the whole dataset.

**Create matrix**

One of the key functions of the STK is to create a matrix for supertree analysis from the input source trees. This function can generate a matrix in a number of formats and also output a single treefile containing all trees in the dataset.

**Safe Taxonomic Reduction (STR)**

A new function for this version is Safe Taxonomic Reduction (STR). This is the only new functionality in this version over the previous version (Davis and Hill 2010). STR is as detailed by Jeffery and Wilkinson (2004), but has been optimised for use on MRP matrices as an assumption is made that the data only contain 0, 1 or ? as characters. This substantially speeds-up computation and can process very large datasets consisting of several thousand taxa, although run times are still days, rather than minutes. STR identifies those taxa that give no extra phylogenetic information and recommends their removal from the matrix. Wilkinson (2001) describes several categories of taxa that the STR algorithm identifies. Of concern are the category "C" taxa where their removal from the matrix is "safe" as they do not provide any additional information, but can be placed back into the supertree post analysis. The STK generates a new matrix as well as a text file detailing the categories of all taxa, as well as identifying those that can be safely removed. In addition, a substitution file can also be generated to put category C* back into the final supertree once generated.

### Funding

KED was funded by BBSRC grant (BB/K006754/1) and a Systematics Association SynTax grant ("Building the arthropod supertree interactively: Malacostracan crustaceans as a test case"2010/11 funding round awarded to Matthew Wills and Mark Wilkinson).

## Web location (URIs)

Homepage: http://supertreetoolkit.org/

Download page: https://launchpad.net/supertree-toolkit/+download

Bug database: https://bugs.launchpad.net/supertree-toolkit/+bugs

## Technical specification

Platform: Linux, Windows, MacOS X

Programming language: Python

## Repository

Type: bzr

Browse URI: https://code.launchpad.net/supertree-toolkit

Location: lp:supertree-toolkit

## Usage rights

### Use license

Other

### IP rights notes

GNU GPL v3

## Additional information

The STK is available from Launchpad (http://launchpad.net/supertree-toolkit). There are two main bzr branches: a stable release version and the development version (trunk). Contributors are expected to branch trunk, develop their new feature and request a merge back into trunk. We encourage all such contributions. STK is released in GPLv3 and is available as source code, via Launchpad's PPA system, as a Windows and MacOS X binary.

A full user manual, including a tutorial and data for the tutorial are available from the Launchpad website.

In future we aim to integrate web-based taxonomy databases to aid taxonomy and nomenclature standardisation. We are also developing a website to release all data that have been collected thus far. The STK will be integrated into that online resource. Finally, we intend to develop a simple tree editing and visualisation GUI such that no external software is required for the whole processing pipeline.

## Supplementary Material

Supplementary material 1 Manual and tutorial data Data type: Mix: PDF, .nex and .phyml
Brief description: The STK User manual and tutorial dataset.
File: oo_6193.zip Jon Hill and Katie Davis
