# Supplementary material for: The Supertree Toolkit 2: a new and improved software package with a Graphical User Interface for supertree construction
Source: Biodivers Data J. 2014 Mar 26;(2):e1053. doi: 10.3897/BDJ.2.e1053 (PMC4031428; doi:10.3897/BDJ.2.e1053)
Supplement: Supplementary material 1 — Manual and tutorial data [file biodiversity_data_journal-2-e1053-s001.zip › SupertreeToolkit.pdf]

Supertree Toolkit Documentation

Release 2

Jon Hill and Katie Davis

February 27, 2014

CONTENTS

1 Introduction **1**
1.1 What is the STK? . . . 1
1.2 What does it do? . . . 1
1.3 What does it not do? . . . 1
1.4 About this document . . . 2
1.5 The processing pipeline . . . 2

2 Getting started **3**
2.1 Introduction . . . 3
2.2 Linux . . . 3
2.3 Windows . . . 3
2.4 MacOS X . . . 3
2.5 Source . . . 4
2.6 Running the STK . . . 5

3 The STK Graphical User Interface **7**
3.1 Starting out . . . 7
3.2 Entering data . . . 9
3.3 Using the interface . . . 10
3.4 Checking data . . . 10
3.5 Processing data . . . 12

4 The STK Command Line Interface **19**
4.1 The basics . . . 19
4.2 Data input/export . . . 20
4.3 Data processing . . . 21
4.4 Miscellaneous functions . . . 22

5 STK Tutorial **23**
5.1 Introduction . . . 23
5.2 Conventions . . . 24
5.3 Collecting Data . . . 24
5.4 Standardising Nomenclature . . . 27
5.5 Removing non-monophyletic taxa . . . 30
5.6 Remove higher taxa . . . 30
5.7 Data independence . . . 32
5.8 Check data . . . 33
5.9 Create matrix or export final tree set . . . 35

6 STK API 37

Python Module Index 43

Index 45

CHAPTER

ONE

INTRODUCTION

1.1 What is the STK?

The Supertree Tool Kit (STK) is software for collecting, curating, storing and processing data ready for inclusion in supertree analyses. It does not build supertrees, however, it does include a number of functions to get the data ready for running a supertree analysis. This includes standardising nomenclature and taxonomy, ensuring adequate taxonomic overlap and creating a matrix. These functions can be used together as a data processing pipeline or independently as stand-alone options for data processing.

1.2 What does it do?

- Import bibliographic files to create a dataset
- Import trees created in most software packages
- Export your data to common formats (Nexus, Newick, Hennig)
- Allow you to summarise your data
- Swap and delete taxa easily
- Create a matrix from your data
- Manage your data graphically
- Ensure data independence
- Ensure adequate taxonomic overlap
- Replace non-monophyletic taxa correctly
- Perform Safe Taxonomic Reduction
- Some post-processing of supertrees (e.g. pruning taxa)

1.3 What does it not do?

- Make supertrees

1.4 About this document

This document is the main manual for the software. Included in the software is context-relevant help embedded in the GUI, which is complementary to the manual. This manual will help you install and use the software, but it will still take experience to know which functions are appropriate for your dataset. In addition to this, many of the functions will require human input e.g. making decisions on how to deal with non-independent data or choosing an appropriate taxonomy.

1.5 The processing pipeline

The idea behind the STK is to create a processing pipeline that is robust, error-free, repeatable and easy. A dataset is created by importing bibliographic data and trees, the STK functions are then used to process these data. Each stage in the pipeline creates a new file, with a history of the previous steps embedded. This way it is easy to undo steps and come back to your data later and understand how it was derived.

Warning: This new file does still need to be saved with a new file name, if you "save as" under the previous saved file you will over-write your previous hard work so be aware!

CHAPTER

TWO

GETTING STARTED

2.1 Introduction

This first chapter is a brief guide to setting-up the STK on Linux, Windows and MacOS X. STK comes as either pre-compiled binaries for some platform or as a source package. Both downloading an archived source package or downloading via bzr are covered. We assume little knowledge of Linux or DOS commands, but some knowledge on how to install software. Contact your local sys admin if you need further help.

We store all source code and downloads on Canonical's Launchpad system: http://launchpad.net/supertree-toolkit

2.2 Linux

The STK is distributed via Launchpad PPA. You need to add the Supertree Toolkit PPA (https://launchpad.net/~stk-developers/+archive/release) to your system, along with an additional one which contains some dependencies. You can then install the package. Run the commands below to do all this.

```
sudo apt-add-repository ppa:fluidity-core/ppa
sudo apt-add-repository ppa:stk-developers/release
sudo apt-get update
sudo apt-get -y install supertree-toolkit
```

This will install the GUI and CLI to standard locations and add the GUI to your Applications menu.

2.3 Windows

A pre-built binary package is available. Simply download and run to install. Follow the on-screen instructions.

The STK CLI is also available from a Command Prompt.

2.4 MacOS X

A DMG is available on Launchpad. Drag the STK icon into the Applications folder. This will install the STK GUI on your Mac.

To install the CLI you must alter your PATH and PYTHONPATH variables. To do this permanently, edit your .bash_profile file and add the following lines:

```
export PYTHONPATH=$PYTHONPATH:\
/Applications/STK.app/Contents/Resources/lib/python2.7/site-packages/
export PATH=/Applications/STK.app/Contents/Resources/bin/:$PATH
```

You can access the STK CLI from a standard terminal

2.5 Source

The source is available as either a compressed tarball or via bzr. To obtain the tarball, simply download from Launchpad, then:

```
tar zxvf supertree-toolkit.tgz
```

Using bzr, you can either obtain the bleeding-edge development version or the current release (recommended) using:

```
bzr branch lp:supertree-toolkit/trunk
```

or

```
bzr branch lp:supertree-toolkit/release
```

respectively.

Regardless of how the source was obtained, you can now either use the STK in-place or install it using:

```
sudo python setup.py install
```

(for Windows users, the sudo is not required).

2.5.1 Prerequisites and dependencies

When running from source you must install the following prerequisites and dependencies:

- Python 2.5 to 2.7
- Matplotlib
- networkx
- libspud
- numpy
- lxml
- pyGTK
- dxdiff (available from http://launchpad.net/spud)

2.6 Running the STK

There are two ways to run the STK: via the GUI (Graphical User Interface) or the CLI (Command Line Interface). Most data collecting and curation is done via the GUI. However, either can be used to carry out data processing. The CLI also contains a few more utility functions that are not available in the GUI.

The GUI is run from the command line using:

```
stk-gui
```

The CLI version is run using:

```
stk
```

which will produce the following help.

```
usage: stk [-h] [-v] [-i]

        {create_matrix,sub_taxa,import_data,export_data,export_trees,export_bib,
         data_summary,safe_taxonomic_reduction,data_ind,data_overlap,permute_trees,
         clean_data,replace_genera,convert_files,create_subset}
        ...
stk: error: too few arguments
```

The STK GUI can also be accessed via the Start Menu (Windows), the Applications folder (Mac OS X) or in the Applications menu (most Linux variants).

CHAPTER

THREE

THE STK GRAPHICAL USER INTERFACE

The Graphical User Interface (GUI) is used to perform all of the STK's primary functionality. It allows you to enter data, visualise data and process data all within a single interface.

3.1 Starting out

To open the GUI either click on the icon or run the following on the command line:

```
stk-gui [file]
```

The GUI looks like this.

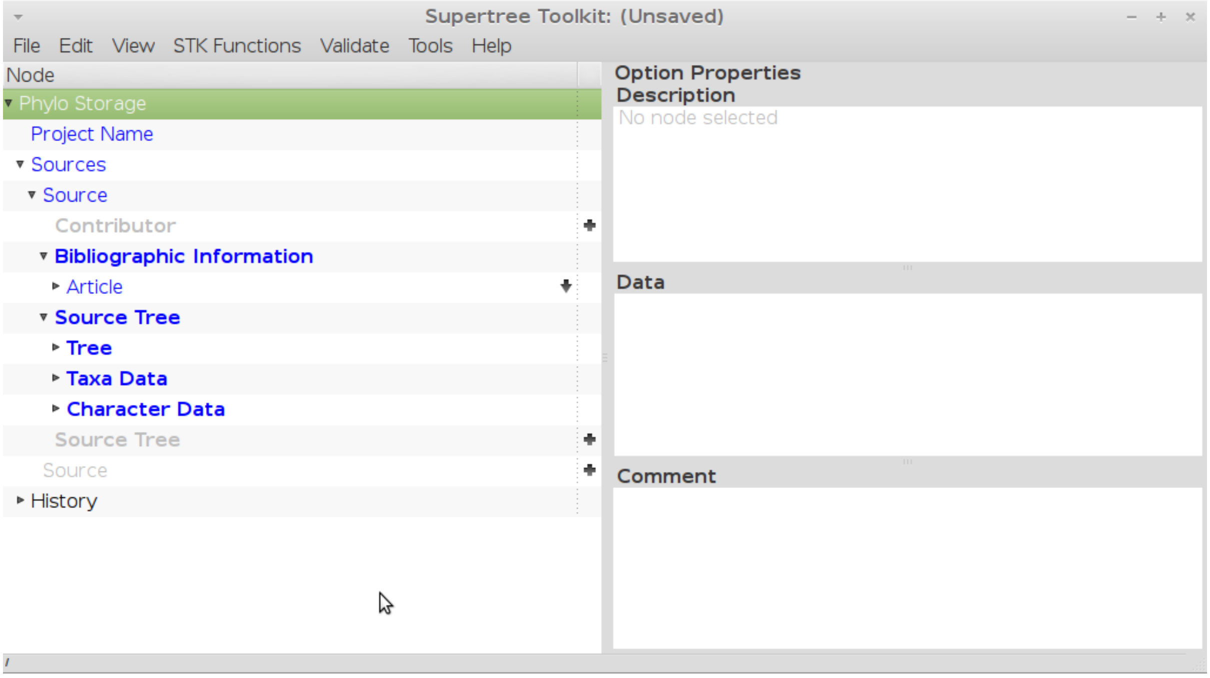

Figure 3.1: The STK GUI with no data loaded. The GUI consists of two vertical panels where data are edited (left) and entered (right).

The GUI consists of two main halves (Fig. 3.1). The left-hand side is a tree-structure that allows you to navigate the data (tree panel). The data are structured into a project, which in turn contains sources,

which in turn contain trees and meta data. The right-hand side (data panel) contains three sub-panels. Each of these divisions is called an element (Fig. 3.2). The top gives context-sensitive documentation on the current selection in the left-hand side. The middle is where you add data. Depending on what part of the data you are editing, the middle panel will change to suit the data to be edited/input. The lowermost sub-panel is where you can add any comments for that part of the dataset. This is not enabled for all sections of the data but should be used wherever it is useful.

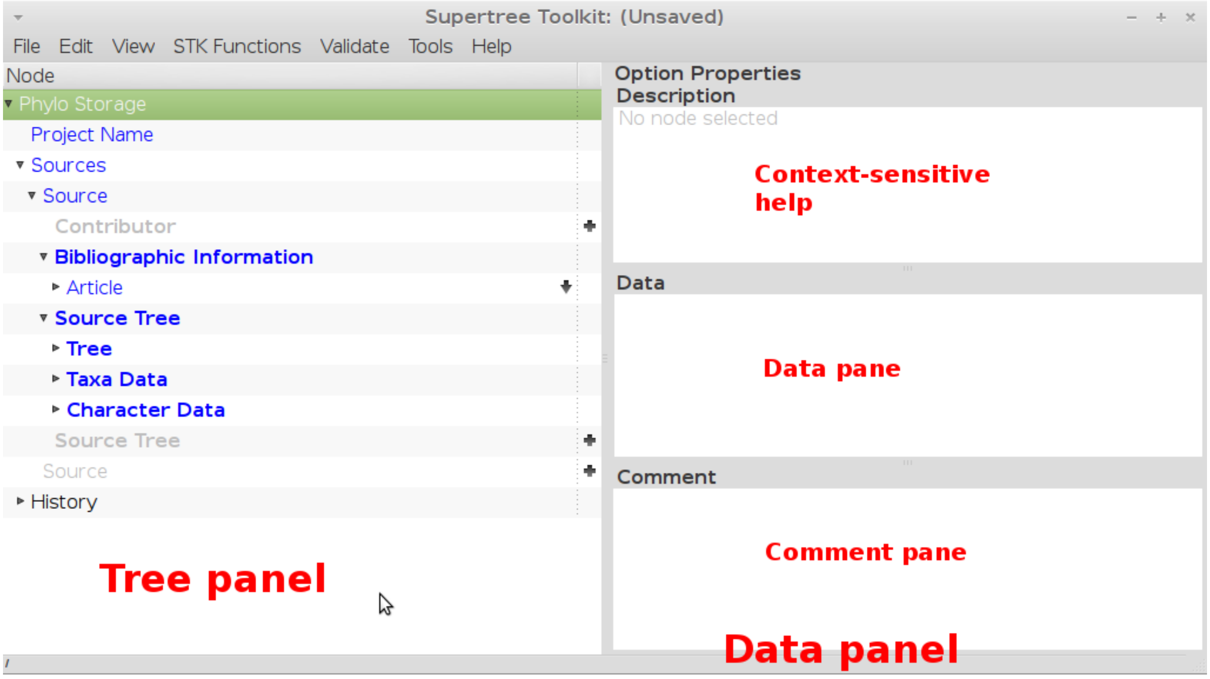

Figure 3.2: The STK GUI with each pane and panel labelled.

To navigate the left hand side, click the small arrows on the left. These will open and close sub-data within the hierarchy. On the right-hand side of the tree, there are small "+" and "-" signs to allow you add or remove data. Where the data are a choice, a dropdown list is activated on the right hand side.

The colour in the left-hand side tree informs you if there is missing data. Blue lines show you are missing required data. The blue then progresses upwards from the missing data, all the way to the uppermost level. This allows you to drill downwards to find the missing data. Black text indicates you have fulfilled the minimum requirements.

There are two types of menus - the main menu on the toolbar and a right-click menu. This toolbar menu contains:

- File
- Edit
- View
- STK Functions
- Validate
- Tools
- Help

Of these, the File and STK Functions are most often used. More on these will be covered later, but briefly the File menu contains commands to open and save data, plus import and export data. The STK

Functions menu contains all the STK-only functionality.

The right click menu allows you to copy and paste elements (e.g. you can copy and past a source from the same or another file) and change how the data are visualised. These are covered later.

3.2 Entering data

The best way to start a new dataset is to import a bibliographic file. The STK uses bibtex format, which is a common format and that all decent reference managers can output, as can most journal websites. We recommend using JabRef, which is free, open source and available on most OS. We have tested the STK extensively with output from JabRef, but your mileage with other reference managers may vary.

Once you have a Bibtex file you can import it using the File->Import from bibliography and import your file. This should import all the papers in that bibtex file and create a source for each one (Fig. 3.3). These are then named in a sensible way and sorted alphabetically. You can then start editing your data.

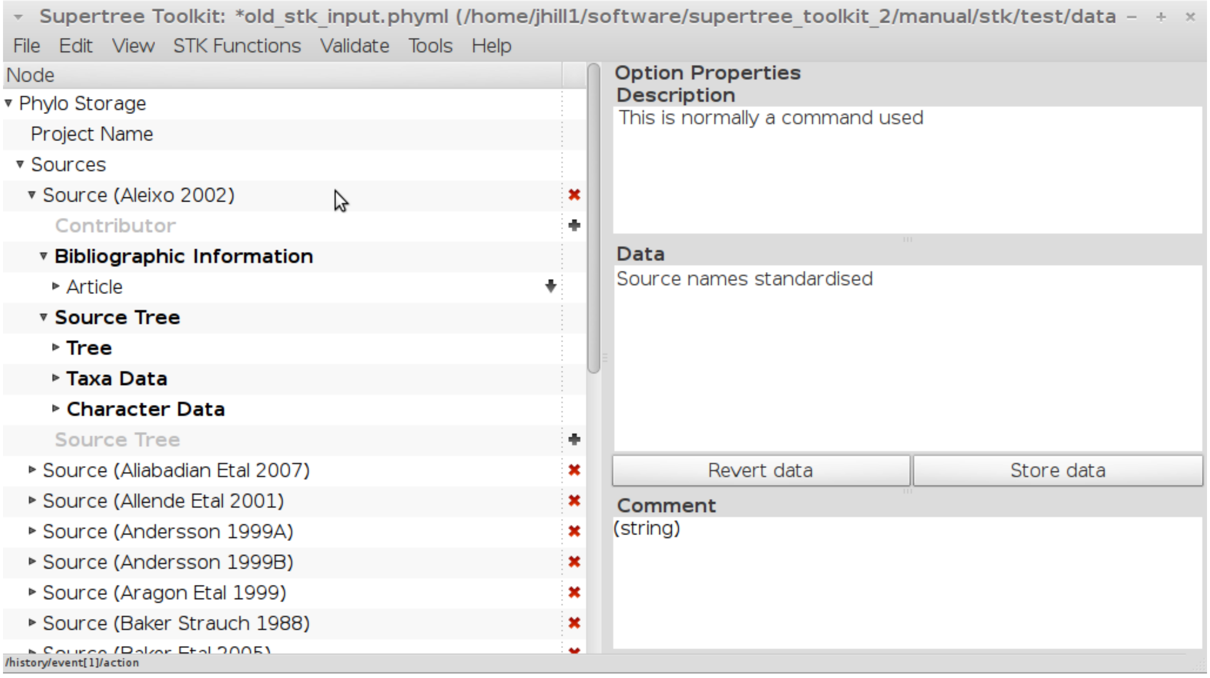

Figure 3.3: A source element that consists of a bibliographic entry, with the data contained in that paper nested below.

The next thing you will want to do is import some trees. You can use any software you wish to digitise your trees. The STK will read the output of most software. To import a tree, drill down the tree panel to the correct source, then open the Tree element and click on the Tree string element. The import tree button will then appear in the status bar, in the lower left of the GUI (Fig. 3.4).

Once done, your tree string will appear in the data panel.

> **Warning:** Avoid non-standard characters in taxa names. Your names *must* not contain commas, parentheses, colons, asterisks, hyphens, slashes or percentage signs (percentage signs are allowed for non-monophyletic taxa - see later). These are not allowed in taxa names in Newick format as they mean other things.

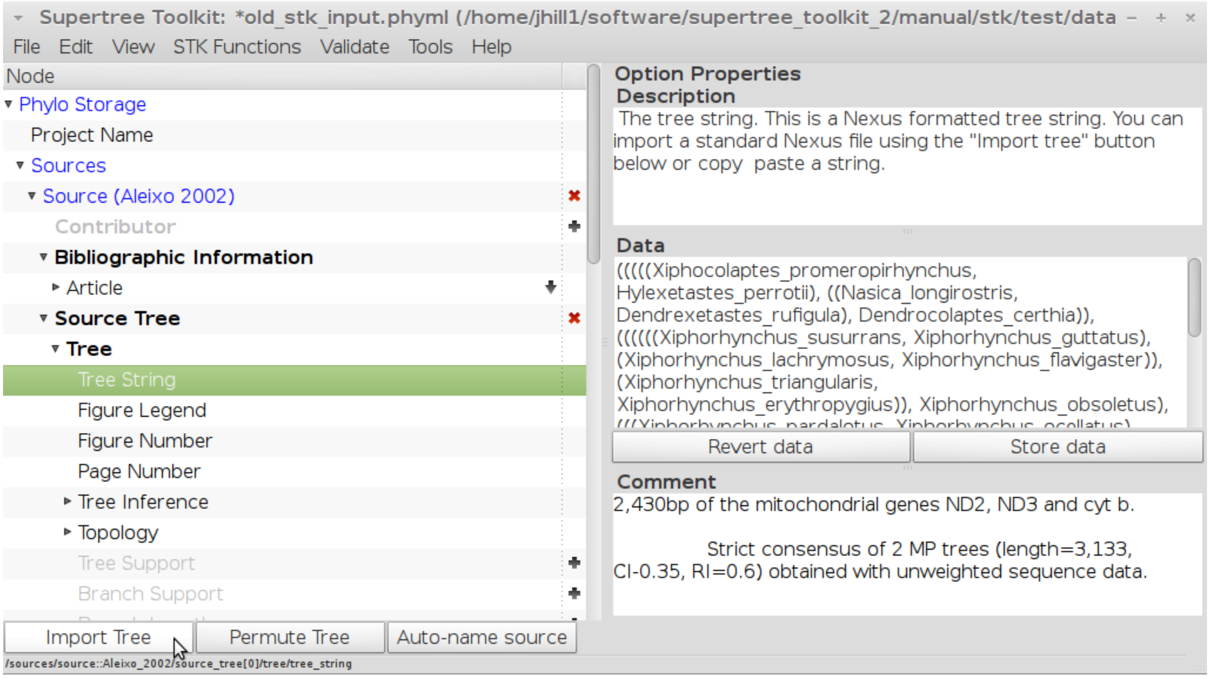

Figure 3.4: The import tree button. Click to import a tree into a source.

Note: Quoted taxa should be done with single quotes only ('), not double or "smart quotes"

3.3 Using the interface

There are a number of useful functions in the STK GUI to aid in data entering and exploration. They are slicing data, grouping data, and copy and pasting data sections.

Grouping data allows all elements of a certain type to be displayed simultaneously. For example, grouping on, say, "Tree String" will show all trees in the dataset. To group data, right click on an element you want to group on and select *Group*. The tree panel will then show the grouping (Fig 3.5). Right-click and select ungroup to return to the original view.

Slicing data allows an easy way to enter similar data on a large number of elements. Right-click page number of any source, select *Slice* and you will see a list of all sources, with the data pane next to each source. You can now quickly edit all page numbers (Fig. 3.6).

Copy and pasting can be done between files or within the same file. Right-click an element, select *Copy*, then select another element *of the same type* and right-click and select *Paste*.

3.4 Checking data

There are a number of functions to help summarise the data and aid in data checking. First is the *Data summary*, which can be accessed via STK Functions->Data Summary. Activating this brings up a window containing the number of trees in the dataset, the taxa list, character list, and years (Fig. 3.7). The output can be saved or copy and pasted as required. This can be used to **carefully** check the taxa list for user errors, for example

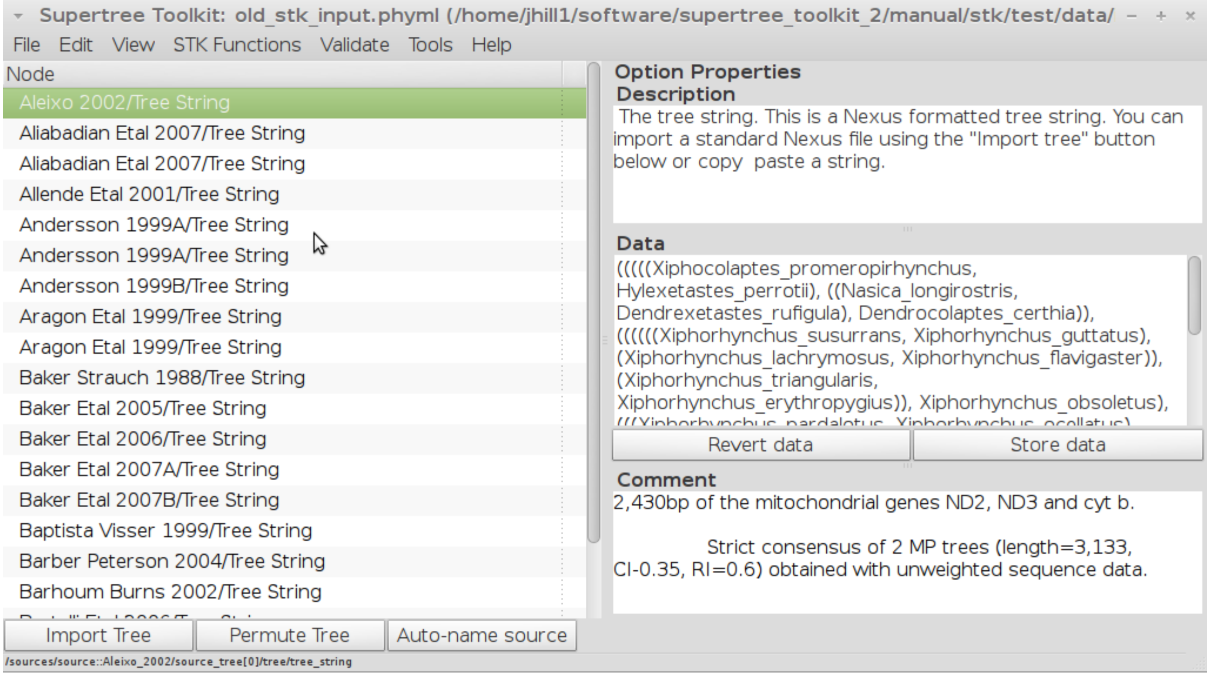

Figure 3.5: Data view after grouping on tree string. Note the source name and all tree strings are all visible.

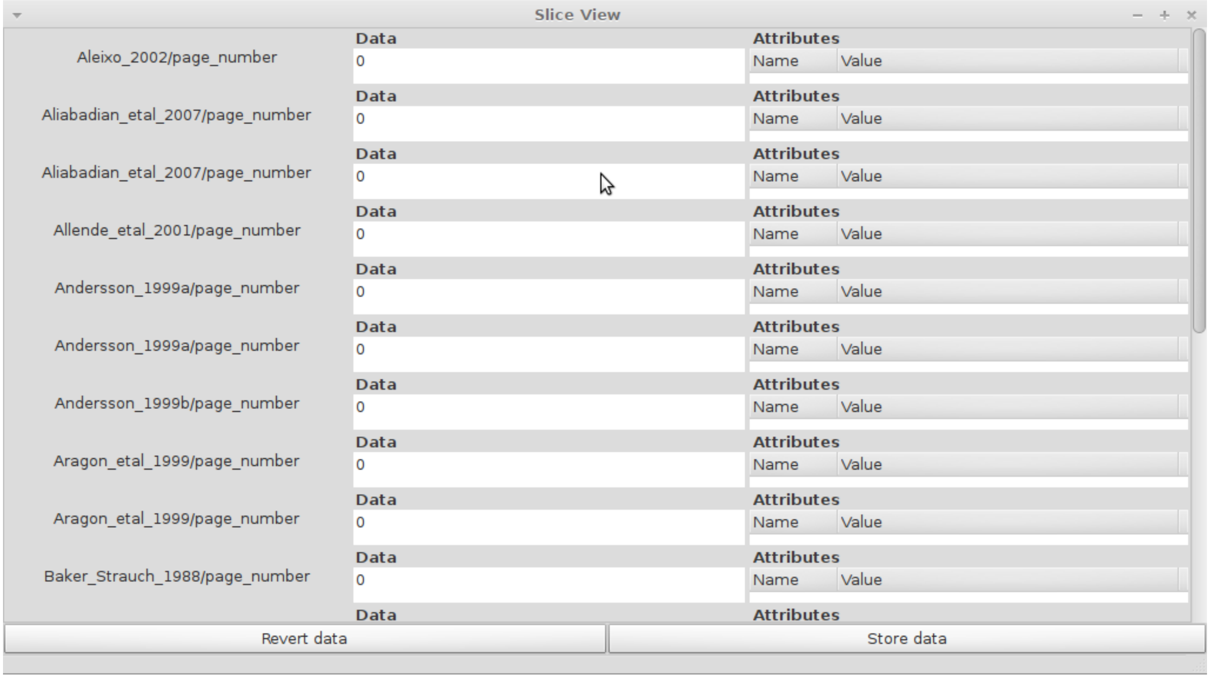

Figure 3.6: Data view after slicing the data on page number.

Note: Incomplete data (with blue elements) may not produce a data summary.

Note: See the tutorial for more information on how nomenclature and taxonomy should be standardised.

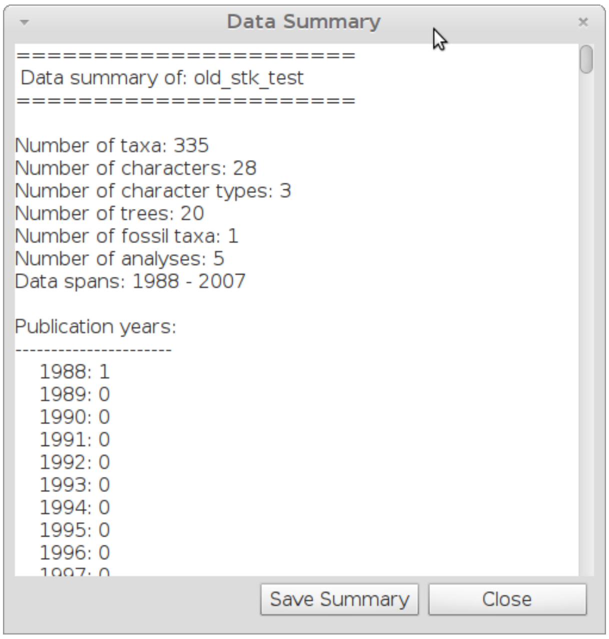

Figure 3.7: Output from the data summary.

Two other functions can also be useful to check the data (and prevent errors when using other functions). *Clean Data* removes non-informative trees. *Standardise source names* ensures all source names are unique and will re-sort the sources alphabetically.

3.5 Processing data

Processing data is done using a number of functions. These are covered in more detail in the tutorial, but briefly compose of the following functions:

- Data independence check
- Data overlap
- Sub taxa
- Permute all trees
- Replace genera
- STR
- Create subset
- Create Matrix

3.5.1 Data independence check

This allows you to check if any of the data in your dataset replicates or is a subset of another data source. The interface shows which sources are identical and can be safely removed in the upper half (Fig. 3.8). The lower half shows subsets. The flagged data should be checked and removed if possible.

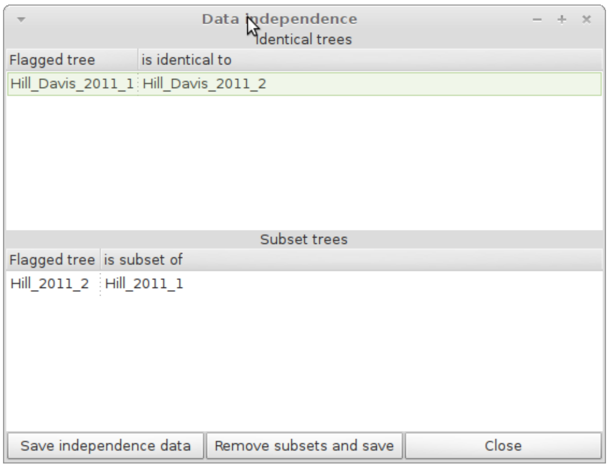

Figure 3.8: Output from the data independence check.

3.5.2 Data overlap

In order to construct a supertree the source trees must have sufficient taxonomic overlap; that is at least two taxa in a source tree must occur in at least one other tree. The STK allows you to both check and visualise this overlap.

The interface (Fig. 3.9) contains options to select the level of overlap (default is 2), which is the number of taxa trees should have in common to be considered connected. The two graphic check boxes will show a window with the result as a graphic. There are two options; the normal graphic (Fig 3.10) and detailed graphic (Fig 3.11).

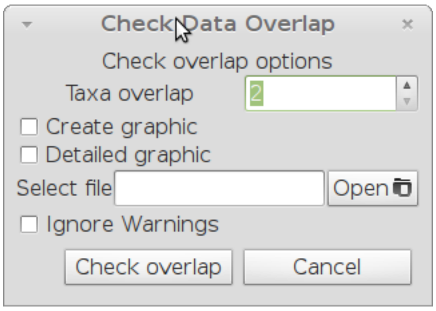

Figure 3.9: Data overlap GUI.

3.5.3 Sub Taxa

Taxa substitutions and deletions are a key part of ensuring a standardised nomenclature and taxonomy for supertree analysis. However, it is usually quite cumbersome to carry out this operation on a number of tree or matrix files. The STK will ensure that taxa substitutions are consistent across the whole dataset

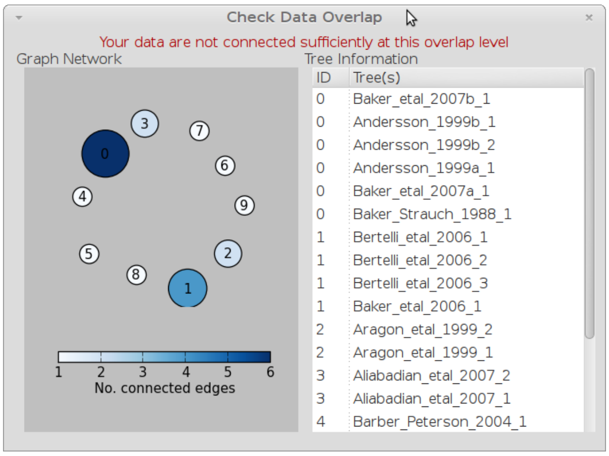

Figure 3.10: Normal graphical view of data overlap. For a correctly connected dataset there should be a single node (circle). These data are not sufficiently well connected.

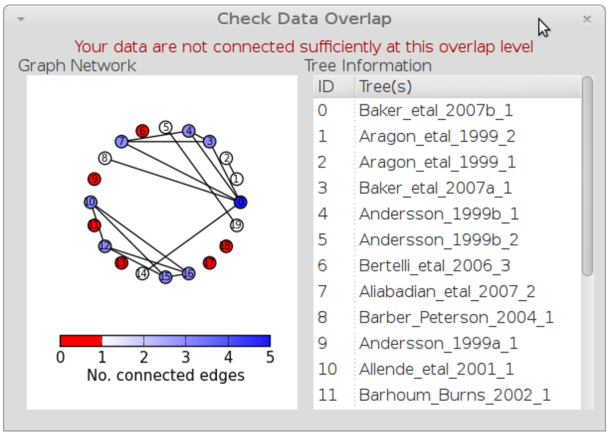

Figure 3.11: Detailed graphical view of data overlap. For a correctly connected dataset there should be no red nodes (circles) in the graph. These data are not sufficiently well connected.

and any taxonomic information is also updated. You can construct taxa deletions and substitutions using the *Sub taxa* interface (Fig. 3.12). Move taxa from the dataset to the right-hand side and add the replacements or leave blank for a deletion. The substitutions created can be saved to a *subs file*. A subs file can also be imported, either as a substitution (or subs) file or as a CSV file.

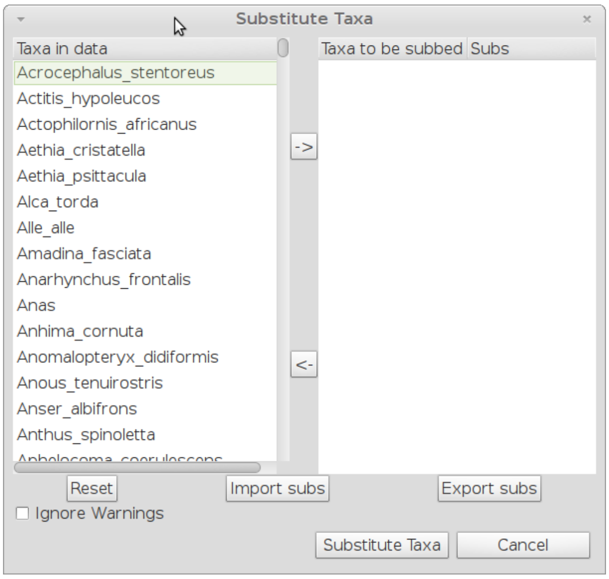

Figure 3.12: Substitute taxa interface. Taxa in the dataset are on the left hand-side. Move taxa to the right-hand side and either leave the Sub column blank for deletions or add a list of taxa.

A *subs file* has the following format:

```
MRPoutgroup =
Dinornithidae = Anomalopteryx didiformis,Megalapteryx benhami
Enantiornithes = Avisaurus archibaldi,Avisaurus gloriae
```

The above file deletes MRPoutgroup and replaces Dinornithidae and Enantiornithes with polytomies of the taxa listed. Deletions cause collapsing of nodes where the deletion occurred.

Note: There *must* be a space either side of the = symbol.

Note that taxa with % signs in the name (see permute taxa below) do not need the % sign in the old taxon name. For example to replace A_a with A_f in the tree:

```
(A_a%1, A_b%1, (A_a%2, A_b%2, A_c, A_d));
```

the subs file should contain:

```
A_a = A_f
```

3.5.4 Permute all trees

When recording trees from the literature inclusions of non-monophyletic can be done using a special encoding of the taxa. Placing a '%' symbol at the end of a taxon name, followed by a number allows the STK to identify these taxa.

To remove non-monophyletic taxa, the tree permutation function is used. This creates a number of trees per source tree, each with a different combination of the non-monophyletic taxa. Note that this produces a tree file containing the unique trees only or a matrix for each source tree in the dataset.

These trees or matrices can then be combined into a single tree using PAUP*, TNT or similar. The consensus of these trees then become the source tree for this source by importing back into the GUI.

3.5.5 Replace genera

Genus-level taxa can be replaced with a polytomy of all species that belong in that genera and exist in the dataset. Replace genera automates this process. It can either create a new Phyml file or a subs file; the latter can be imported into the Sub taxa function.

3.5.6 STR

Safe Taxonomic Reduction identifies possible problem taxa in the dataset, which may cause instabilities in the supertree analysis. The output files from STR are (Fig. 3.13):

- Subs files for deletion and replacement of appropriate taxa (optional)
- A text file containing the STR output. This contains lists of taxa in the A, B, C, D, and E categories. Note that this file can be very large for datasets containing hundreds of taxa.

Note: This can take a long time for even small datasets. For anything over 100 taxa use the command line interface.

For further details on STR see Wilkinson (1995).

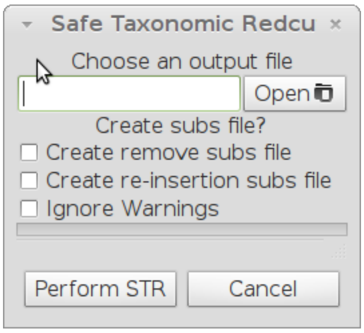

Figure 3.13: STR interface. The output file contains the equivalency matrix. The two optional sub files will automatically allow deletion and reinsertion of taxa where this is safe to do so.

3.5.7 Create subset

You may want to create a subset of your entire dataset, based on, say, year of publication, characters used or taxa included. The create subset allows you to define criteria on which this subset can be created. The GUI (Fig. 3.14) allows you to create this search by clicking "Add". Then select the criterion from the drop-down list on the left hand side. Then in the right hand column, enter your terms. For years you can enter a range, like 2000-2010. For all terms, a comma separated list can be used to enter multiple terms (Fig. 3.15).

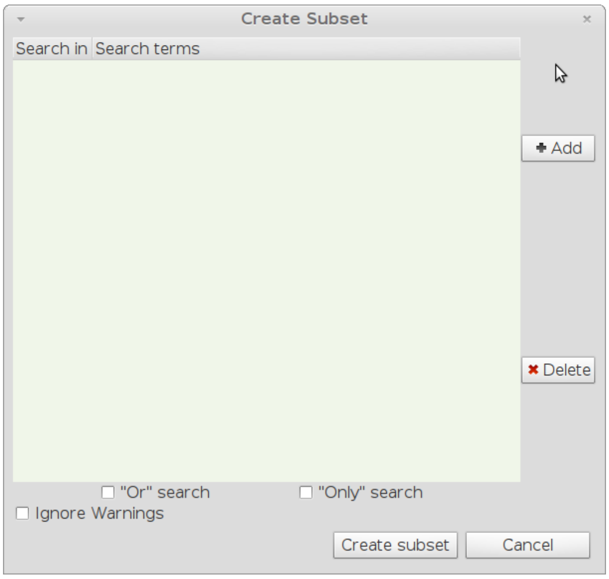

Figure 3.14: The initial create subset interface. The two buttons on the right allow you to add and delete terms. Terms appear in the main part of the window. The left side of this contains a drop down list to select the criterion. The right hand side is where you enter terms.

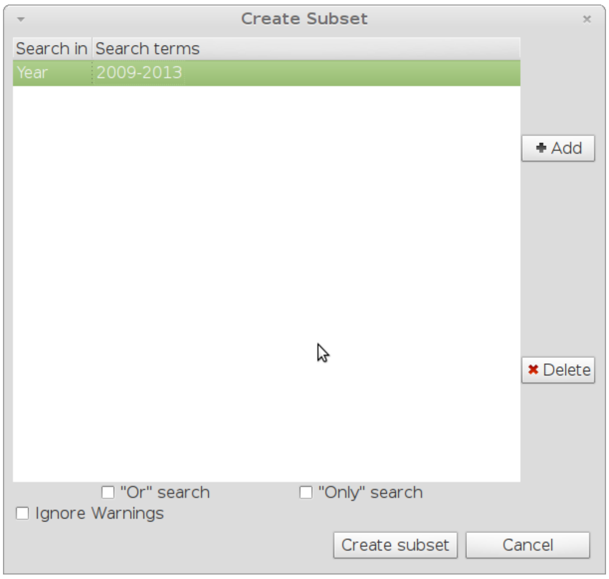

Figure 3.15: A search for data published between 2009 and 2013 has been set up.

Once complete, a search will ask you to save the output to a new Phyml file.

Warning: This new file does still need to be saved with a new file name, if you use the same filename as the existing file you will over-write your previous hard work so be aware!

3.5.8 Create matrix

After all your processing, the final step is to create a matrix of your data. This function will create a matrix suitable for reading into PAUP**, TNT and most other supertree software. Note that some software require a set of "input trees". In this case, use the "Export trees" function under the the "File" menu. Matrices can be output in Nexus or Hennig (TNT) format. Simply select "Create matrix", choose your options, including a filename, and click create matrix. This might take a few minutes for large datasets. You can also add a taxonomy tree and optionally ignore outgroups that have been specified for each tree.

Weights will only be used in TNT format and are applied automatically. Remove any "weights" elements from your data if you do not wish to use weights.

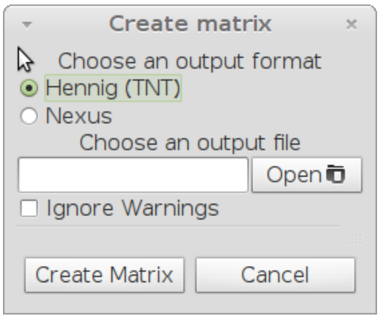

Figure 3.16: The create matrix format. Select your options and click "Create matrix".

CHAPTER

FOUR

THE STK COMMAND LINE INTERFACE

The STK CLI contains a number of functions for initialising and processing data.

4.1 The basics

The CLI is initialised using:

```
stk
```

This will produce:

```
usage: stk [-h] [-v] [-i]

        {create_matrix,sub_taxa,import_data,export_data,export_trees,export_bib,
         data_summary,safe_taxonomic_reduction,data_ind,data_overlap,permute_trees,
         clean_data,replace_genera,convert_files,create_subset}
        ...
stk: error: too few arguments
```

The STK has a number of commands relating to data input and export, processing, and some miscellaneous functions. These are detailed below.

To run a command, e.g. the create matrix function, the command is:

```
stk create_matrix
```

This will produce the help for the create matrix function:

```
usage: stk create_matrix [-h] [-f {hennig,nexus}] [--overwrite] input output
stk create_matrix: error: too few arguments
```

More detailed help can be obtained using the ‘’‘-h’‘’ flag.

```
>$ stk create_matrix -h
usage: stk create_matrix [-h] [-f {hennig,nexus}] [--overwrite] input output

positional arguments:
    input                 The input phyml file
    output                The output matrix file

optional arguments:
```

```
  -h, --help            show this help message and exit
  -f {hennig,nexus}, --format {hennig,nexus}
                        The format of the matrix. hennig or nexus. Default is
                        hennig
  --overwrite           Overwrite the existing file without asking for
                        confirmation
```

The options can be given with either the long format (–help) or (-h). Not all arguments have both (e.g. –overwrite).

Note the stk itself has three options:

- -h – help
- -v – verbose
- -i – ignore warnings

Note that these *must* come before the function you want to use. For example this is valid:

```
stk -v create_matrix
```

This is not.

```
stk create_matrix -v
```

The functions are divided into input/output and processing, with one additional miscellaneous function used for converting data files. Below is a brief description of each function. Use the ‘‘-h’‘ flag for information on options and further details of input/output for the function.

4.2 Data input/export

4.2.1 export_bib

Exports a bibliographic file containing the references for all your sources. This output is a standard bibtex file.

4.2.2 export_data

Exports the data to the old STK format. This is directory based, with each source in a separate directory. The sources are split into two files per tree: an XML data file containing the meta-data and a tree file.

4.2.3 export_trees

Export all the trees in the dataset into a single tree file.

4.2.4 import_data

Import data from the old STK format into a Phyml. Note there may be issues with author names which should be in the format of “FirstName1 LastName1 and FirstName2 LastName2”.

4.3 Data processing

4.3.1 clean_data

Remove all non-informative trees and sources from the dataset. These are trees that contain only three or fewer taxa.

4.3.2 create_matrix

Create a Hennig or Nexus matrix using Baum and Ragan coding of all trees in the dataset.

4.3.3 create_subset

Create a subset from your data, specifying various criteria, including year published, characters contained and taxa included.

4.3.4 data_ind

Check your data for adequate data independence. The output is a CSV file that can be opened in a standard spreadsheet package and contains identical and subset categories. It can also give you a new Phyml with non-independent data removed.

4.3.5 data_overlap

Check your data for adequate taxonomic overlap. Optional extras are graphical outputs.

4.3.6 data_summary

Produce a text summary of the data, containing a taxa list, character list and other useful things.

4.3.7 permute_trees

Permute individual trees or all trees containing non-monophyletic taxa (indicated by a '%' symbol). Output is tree file or matrix for analysis.

4.3.8 replace_genera

Replace all generic level taxa with a polytomy of all species of that genus already in the dataset.

4.3.9 safe_taxonomic_reduction

Perform safe taxonomic reduction on the dataset. Output is the equivalency matrix, plus the option to give subs files to safely delete and re-insert taxa

4.3.10 sub_taxa

Substitute or delete taxa from the dataset. Returns a new Phyml.

4.4 Miscellaneous functions

4.4.1 convert_files

Convert a tree file or matrix into Nexus, Newick (tree only) or Hennig (matrix only) formats.

CHAPTER

FIVE

STK TUTORIAL

5.1 Introduction

The following is an example of how the scripts were used in creating a species-level supertree of Anomura - an infraorder of decapods.

There are several files included in the tutorial dataset:

- A bibtex file containing all the original bibliographic datasets
- A semi-complete Phyml with a single tree missing
- A tree file to import into the above
- The completed original dataset
- The complete processed dataset
- The final matrix, ready for supertree construction
- A supertree, generated using TNT

Note: This is a test dataset, which has been amended to show all the features and functions of the STK. Do not use these data in a real analysis.

The aim of this tutorial is to guide you through the stages of collecting, storing and curating supertree source data (Fig. 5.1). This can be divided into a number of steps:

- Collect and import bibliographic data
- Collect, digitise and import trees
- Standardise nomenclature (remove synonyms, misspellings, vernacular names, etc.)
- Deal with non-monophyletic taxa
- Standardise taxonomy (e.g. remove higher level taxa)
- Check data independence
- Check taxonomic overlap
- Create a subset
- Create a matrix

In carrying out this tutorial, you will cover most of the functions of the STK.

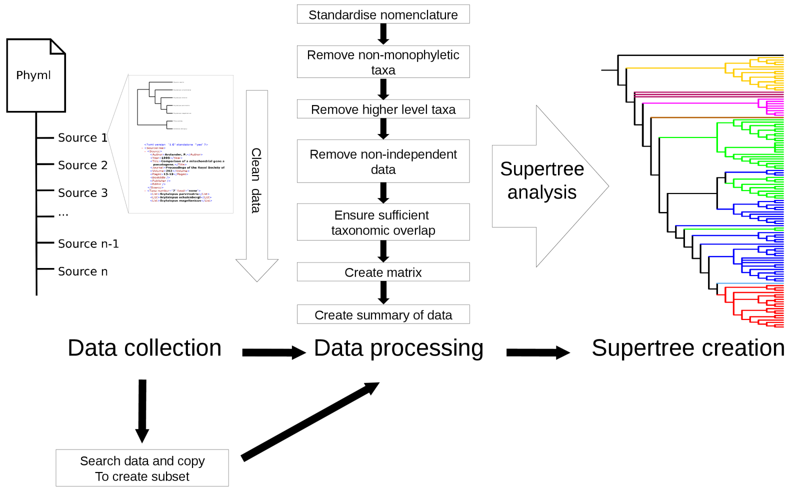

Figure 5.1: An illustration of the pipeline that can be created using the STK processing functions.

5.2 Conventions

The bulk of the tutorial can be done using either the GUI or the command line. Command line instructions are denoted by:

an example command

GUI instructions are denoted by:

Start → *Programs*

Filenames are given by the following:

`an_important/file.phyml`

There are also notes along the way, which are shown like this:

Note: These are hints and tips

Finally, some warnings are also given:

Warning: This is a warning

5.3 Collecting Data

Data collection occurs in two stages: literature collection and tree digitisation.

Literature collection is carried out searching for relevant taxonomic terms in conjunction with terms such as "phylog*" in order to obtained literature containing original phylogenetic trees. Bibliographic data is stored in Bibtex format, as the STK can import Bibtex files directly. Bibtex is a common format and

all decent reference managers can output, as can most journal websites. We recommend using JabRef, which is free, open source and available on most operating systems. We have tested the STK extensively with output from JabRef, but your mileage with other reference managers may vary.

Once all bibliographic data are collected they can be imported into the STK to provide the basic information for your dataset.

Open a new instance of the GUI by double clicking the installed icon, or typing stk_gui at a terminal. Using the menus, go to *File* → *Import from bibliography*. Use the GUI dialog to navigate to the Bibtex file `tutorial/bibliography.bib` and open it. You will now see a list of sources in the left hand side of the GUI. Note that all sources appear blue as there is a lot of missing meta data that needs completing.

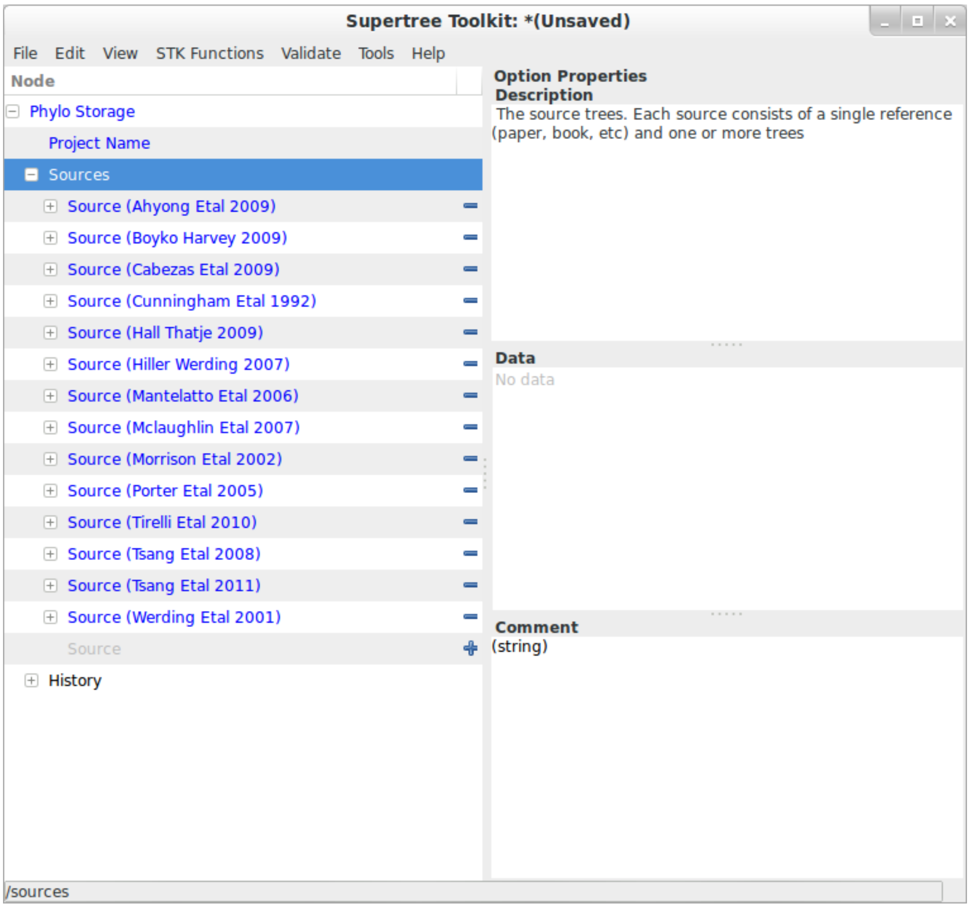

Figure 5.2: The result of importing the bibliographic file included in the tutorial. Note the blue colour, which means there is missing data (the trees and associated metadata).

The next step is to digitise your trees and import them. We've already done this, so open `tutorial/starting_data.phyml` which will have the bibliographic data and all but one source tree completed.

You can practice digitising trees using Treeview, Mesquite or similar software.

> **Warning:** Avoid non-standard characters in taxa names. Your names *must* not contain commas, parentheses, colons, asterisks, hyphens, slashes or percentage signs (percentage signs are allowed for non-monophyletic taxa - see later). These are not allowed in taxa names in Newick format as they mean other things.

Note: Quoted taxa should be done with single quotes only ('), not double or "smart quotes"

The tree missing from the dataset is shown below.

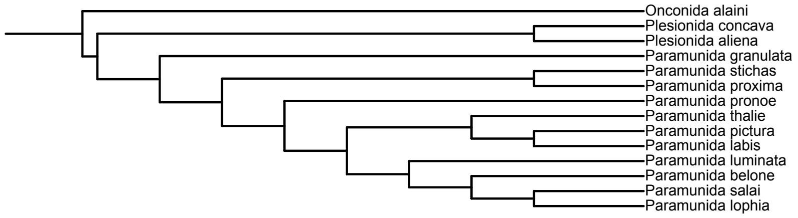

Figure 5.3: This is the tree missing from the dataset

To add the remaining source tree, navigate the source to reach Cabezas et al 2009 (this is easy as it's the only blue source highlighted). Drill down to reach the tree_string entry (again, the only blue one). On the lower left of the GUI, click *Import tree* and navigate to the tree file `tutorial/Cebezas_etal_tree1.tre` (or use your own digitised tree). This should place the tree file into the GUI.

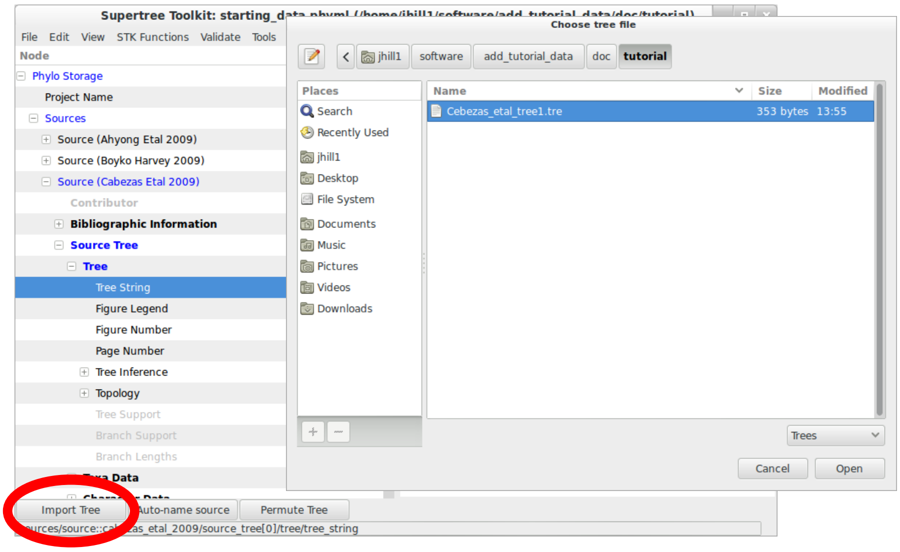

Figure 5.4: Using the blue colour as a guide, navigate to the source with missing tree. Click *Import tree* (circled in red) and load the tree.

Now save your Phyml using the *File* → *Save As* and type in a name (suggest `Anomura.phyml`).

Note: Once done, this is your original file before any processing. Keep this safe. When you extend or alter the data later, you should begin with this file.

It is worth noting at this point that non-monophyletic taxa need some special attention. The STK allows you to *permute* the positions of these taxa and generate a tree with all possible combination of places of the taxa. These permuted trees can then be dealt with later. However, you must be aware of this when digitising trees. To indicate a taxon is non-monophyletic append a '%d' on the end of the name where d is an integer. For example, Fig. 5.5 can be encoded as:

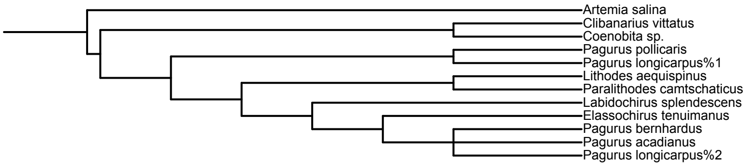

Figure 5.5: Non-monophyletic clades can be denoted with %n in the name as above.

```
(Artemia_salina, (((Pagurus_pollicaris, Pagurus_longicarpus%1),
((Labidochirus_splendescens, (Elassochirus_tenuimanus, (Pagurus_bernhardus,
Pagurus_acadianus, Pagurus_longicarpus%2))), (Lithodes_aequispinus,
Paralithodes_camtschaticus))), (Clibanarius_vittatus, Coenobita_sp.)));
```

You can see such a tree in the tutorial dataset in Cunningham et al 1992. We will see how to permute these trees later in the tutorial.

At this point it's worth creating a data summary – this will allow you to spot data input errors: typos, copy and paste errors, etc. Execute the data summary command using the GUI or command line:

STK Functions → Data Summary

stk data_summary -d summary.txt Anomura.phyml

Carefully check the output for errors. However, it is important not to correct "errors" that exist in the original paper – these are dealt with later. However, the data summary will allow you to spot where you might have mistyped a character (CYtb instead of Cytb, for example) or didn't quite copy and paste the taxa correctly (missing the last few characters for example). All lists are sorted alphabetically, which makes spotting these kinds of errors relatively straightforward.

There are other basic *housekeeping* tasks that can be useful at this point too. First, standardising the source names using *STK Functions → Standardise source names* to ensure each source has a unique name. Second, cleaning the data using *STK → Clean data* to remove all uninformative trees and remove non-monophyletic taxa where only one possible combination exists.

The above tasks will not alter the tutorial data, so it's safe to save the file again. Normally you would use *File → Save As* to be sure of not overwriting data. Note that the file has altered though. If you navigate to the *History* section you will see that the data summary and clean data commands have been recorded, along with the date and time. This enables you to track what commands have been run on this Phyml dataset.

5.4 Standardising Nomenclature

Warning: From this point on we will create a new file for each step of the process. This is good practice in case of user or software errors. Take note of the filename changes as we process the data.

The next stage is to standardise the taxa - removing synonyms and higher taxa.

Note: The tutorial dataset has a sub file already defined. Below is for information only.

Removing synonyms requires that a "standard" taxonomy is used. It does not matter what this is, but it does matter that two taxa that are actually the same taxa have the same name to avoid artificial inflation of the taxa number and also to improve overlap between the source trees. Services such as ITIS, WORMS,

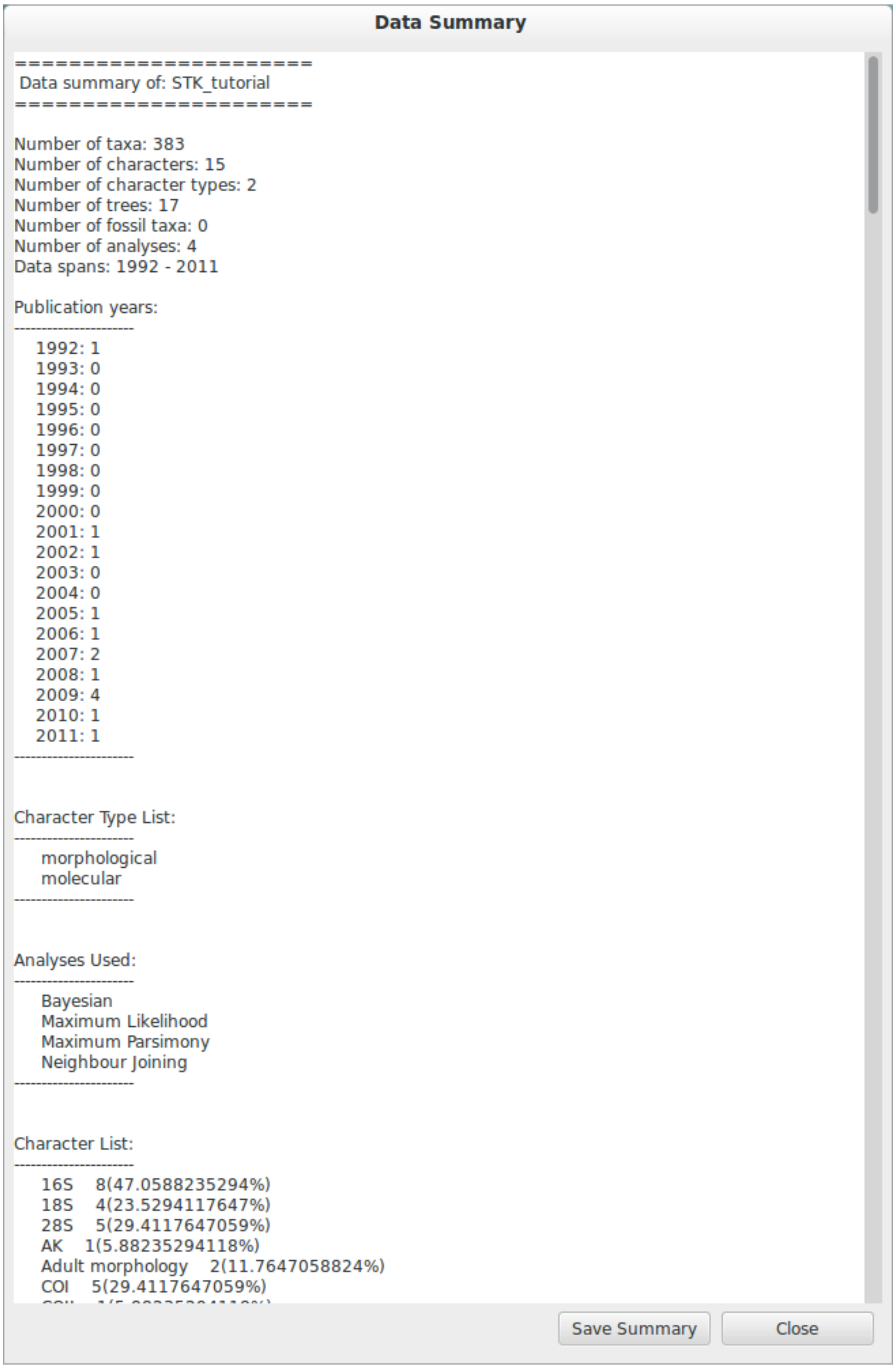

Figure 5.6: Example data summary.

Encyclopedia of Life and other online, specialised, databases are useful. In future the functionality of creating a standardised taxonomy is planned to be included in STK. Once a standardised taxa has been decided, the names can be replaced.

Use your taxonomy to create a *subs file*. This can be done manually in a standard text editor or using the STK GUI. A subs file is a simple text file where taxa equivalency is denoted. Using a text editor, create a file like this one:

```
Aegla denticulata denticulata = Aegla denticulata
Axius vivesi = Neaxius vivesi
Calcinus tibicen = Gilvossius setimanus
Callianqssa tyrrhena = Callianassa tyrrhena
Cambarus bartoni = Cambarus bartonii
Cliopagurus galzini = Ciliopagurus galzini
```

Note that spaces can be replaced with underscores if needed, but spaces must occur *both* sides of the '=' sign. The above is an excerpt from the subs file included in the tutorial dataset, which replaces a sub-species and corrects some common misspellings and synonyms.

Alternatively, create a simple CSV (Comma Separated Value) file in Excel or similar. The first column contains the taxa already in the dataset and the subsequent columns are the taxa to be substituted into the dataset. Each substitution is on a new row. Ensure you save the file as a Comma Separated Value (CSV) file.

The above can be created using the GUI which ensures you only add taxa already in the dataset on the left-hand side. Using *STK Functions → Sub taxa*, you will be presented with the interface to create your substitutions.

Move taxa from the left to the right using the arrows. Then double-click the second column on the right-hand side and add the taxa to be subbed to this column. Using the subs defined above, the GUI will look like this.

Note you should export the substitutions at this point into a subs file, which you can import back into the interface at a later date.

Once you have a *subs file* you can replace the taxa. Using either the GUI or the command line run the sub taxa function on your Phyml. In the GUI, import your subs file (or CSV file) and, fill in a new filename and click *Sub taxa*. For the CLI, run this command:

stk sub_taxa -s subs_file input.phyml output.phyml

This replaces and deletes the taxa defined in your *subs file* in all trees in your dataset.

For our tutorial dataset, we have already created the subs file for you. Run this on `Anomura.phyml` using the GUI or command line:

stk sub_taxa -s standard_taxonomy.dat Anomura.phyml Anomura_subbed.phyml

In the GUI use *STK Function → Sub taxa* and then *Import subs* to import the subs file. Then click *Substitute Taxa*. Give the filename `Anomura_subbed.phyml` and click save. This will give you a warning message. This is fine, so click OK (we want to put in new taxa). You'll get confirmation the substitutions have been successfully carried out and saved to a new file. Now save the currently open file (`Anomura.phyml`) as a new *history* entry has been added, containing details of the substitution. You now have *two* files: your original with an additional history event detailing the substitutions done (`Anomura.phyml`), and a new file where the substitutions have taken place, including a history event stating how the file was created (`Anomura_subbed.phyml` or whichever name you saved as).

5.5 Removing non-monophyletic taxa

To remove non-monophyletic taxa, the tree permutation function is used. As mentioned above, non-monophyletic taxa are dealt with separately and denoted with a '%n' in the taxon name where n is an integer. We deal with these taxa by permuting every possible location of these taxa. This creates a number of trees per source tree, each with a different combination of the non-monophyletic taxa. Note that this produces unique trees only. These can then be output in a single tree file or as matrix. You take this and create a 'mini-supertree' which becomes your single source tree. For example load into PAUP* or TNT and get the tree required with a branch-and-bound search or heuristic search for larger trees.

There is one tree in our test dataset that requires removal of non-monophyletic taxa. Create a matrix using either *STK Functions* → *Permute all trees* (call the output `anomura_poly.tnt` and use Hennig format) or use the command:

stk permute_trees -c hennig Anomura_subbed.phyml Anomura_poly.tnt

The above command will create a matrix for each permutable tree (in this case one matrix) which will be called `anomura_poly_cunningham_etal_1992_1.tnt`.

Run this matrix in TNT to generate a mini-supertree. The commands below are suggestions for how to do this in TNT.

```
run anomura_poly_cunningham_etal_1992_1.tnt;
ienum;
taxname=;
tsave *permuted_cunningham_etal_1992.tnt;
save;
tsave /;
nelsen*;
tsave *permuted_cunningham_etal_1992_strict.tnt;
save /;
tsave /;
quit;
```

You can then re-import this tree into your dataset, replacing the original tree with the strict consensus `permuted_cunningham_etal_1992_strict.tnt`. Navigate to Cunningham_et_al_1992 and replace the tree with the % symbols in the taxa name by clicking *Import tree*. Now *File* → *Save as* to filename `Anomura_poly.phyml`.

5.6 Remove higher taxa

Our dataset currently contains vernacular names and higher-order (e.g. family) names. These have to be removed and replaced with polytomies. As this must happen each time a supertree is produced, it is best done with via a taxa substitution file. You can create this file once, amend as appropriate and run each time you alter the data before supertree analysis is done. For example:

```
Albuneidae = Albunea,Austrolepidopa,Harryhausenia
```

replaces any source tree containing the higher order taxa *Albuneidae* with polytomies.

We can replace using genus or species names. When replacing with genera, species will be replaced in a later step. Therefore, it is recommended you make your substitution file as comprehensive as possible. You can then keep it for later when you extend the dataset. Note that the species listed should be in the dataset already, but you can avoid thoroughly checking this as you can use the "replace existing taxa

only” option in the replacement. When replacing with genera this is not necessary. You can use the data summary output to check how well these substitutions have worked.

Once your substitution file is ready, you can use either the GUI or CLI to replace taxa in a Phyml. The output of this is a new Phyml with the taxa replace or deleted as dictated in your subs file.

The command line would be:

stk sub_taxa -e -s SUBFILE input.phyml output.phyml

To use the GUI, simply clicking *STK Functions -> Sub Taxa*, loading your subs file, and clicking *Sub taxa*.

Note: It is important here to only substitute in *existing taxa* so use the -e flag on the CLI and click the *Only existing taxa* in the GUI if you are substituting in species to avoid adding extra taxa.

Finally, to guard against errors and bugs, back-up your data ‘‘‘before’‘‘ carrying each set of substitutions. If you come across something that went wrong, report a bug on our Launchpad. Replacing taxa in trees is not straightforward at times so this is definitely the time to check your backups.

Our Anomura data have one such higher taxa and we have introduced an extra taxon by creating the mini-supertrees earlier: MRP_Outgroup. Carry out a data summary on `Anomura_poly.phyml` and you should see the MRP_Outgroup and *Albuneidae* in the list. We therefore need to create a simple subs file using one of the three possible ways (CSV, subs or via the GUI) such that we have the following substitutions (below is in subs file format):

```
Albuneidae = Albunea,Austrolepidopa,Harryhausenia
MRPOutgroup =
```

which will delete the taxon.

Note: There are two spaces either side of the ‘=’ for the MRP_Outgroup

---

In the GUI, use *STK Functions* → *Sub taxa* to move *MRP_Outgroup* from the left to the right of the interface; likewise for Albuneidae. Leave the second column blank for MRP_Outgroup, but fill in *Albuneidae* with *Albunea,Austrolepidopa,Harryhausenia*. Then click *Substitute taxa* to do the substitutions. Save the file as `Anomura_no_higher.pyml`.

On the command line use the following command to delete the *MRP_Outgroup*:

stk sub_taxa -o MRP_Outgroup Anomura_poly.phyml Anomura_no_higher1.phyml

which will delete the taxon. Then do (on a single line):

stk sub_taxa -o Albuneidae -n “Albunea, Austrolepidopa, Harryhausenia” Anomura_no_higher1.phyml Anomura_no_higher.phyml

To do the replacement of *Albuneidae*. Note that we have not needed a subs file when using the CLI for this trivial substitution.

5.6.1 Replacing genera

The final part of this process is to replace all genera with their constituent species that are already present in the dataset, e.g. *Aegla* is replaced with a polytomy of all species belonging to *Aegla*. This is done with

the replace genera function. Only species already in the dataset are added. This is a similar function to the general substitute taxa functions, but it generates the substitutions for you.

To run this you can either use the GUI or CLI. The CLI command is:

stk replace_genera Anomura_no_higher.phyml Anomura_species.phyml

In the GUI, use *STK Functions → Replace genera*. Get the STK to create a new Phyml for you, named `Anomura_species.phyml`

Note: This is the "standard" data - *keep this* as this is what gets updated when new trees are added to the dataset.

The next few steps need doing each time you need to generate a supertree after adding more source data and have re-standardised the taxa

5.7 Data independence

This is the first step that is needed each time a tree is generated. We need to check for data independence, remove vernacular and higher names.

The data independence check is done via the data independence function. The function checks if any source meets the following conditions:

- Uses the same characters
- *and* is either a subset of, or contains the same taxa as, another source.

If these two conditions are met, the two sources are not independent. If the two sources are identical (same taxa and same characters) it is up to you which one is included, or you can create a mini-supertree of them to create a single source. When one source uses the same characters but is a taxonomic subset of another, you should include the larger source tree. The data independence function places source trees into these two categories and informs you of the equivalent source. You can then simply delete sources as required using the GUI. The STK can automate most of this process (but do check the result to make sure you agree).

Using the command line, type the following:

stk data_ind Anomura_species.phyml -n Anomura_ind.phyml

This will create a new Phyml with all non-independent *subset* data removed, using the above rules. Trees that are identical will not be removed. You have to decide which one should be removed or combine them using a mini-supertree. The same can be achieved in the GUI using the *STK Functions → Data Independence Check* and clicking *Remove subsets and save*, giving `Anomura_ind.phyml` as the filename.

To deal with identical data, open a new STK GUI and give it a temporary name. Then copy and paste the sources that contain the identical trees from your existing dataset into your new one. You can delete any trees that aren't identical but were copied over at this point. You can now make a matrix using *Stk Functions → Create Matrix* and create a supertree.

For our tutorial dataset we have the following non-independent data:

```
Source trees that are subsets of others
Flagged tree, is a subset of:
boyko_harvey_2009_1,mclaughlin_etal_2007_1
```

```
Source trees that are identical to others
Flagged tree, is identical to:
Ahyong_etal_2009_2,Ahyong_etal_2009_1
```

So, running

stk data_ind Anomura_species.phyml -n Anomura_ind.phyml

or via the GUI, you can remove Boyko and Harvey 2009, tree 1 manually or use the *STK Functions* → *Data Independence Check* and clicking *Remove subsets and save*, giving `Anomura_ind.phyml` as the filename.

> **Warning:** If you removed the source manually, remember to "Save as"

To deal with the two identical trees, open a new STK GUI and copy and paste the Ahyong_etal_2009 across. This source only contains those two trees, so simply create the matrix using *STK Functions* → *Create Matrix*. Run this matrix in TNT (see above for example commands) to create a combined source tree to import back into your original (`Anomura_ind.phyml`) file

In `Anomura_ind.phyml`, remove one of the Ahyong_etal_2009 source trees and import the output from TNT into the other. It is advisable here to edit the figure legend etc. to match that this is now a combined tree (in this dataset the figure legend etc. contain dummy data) and to add a comment on this tree with the TNT commands used as a reminder in future of where this tree came from. Save this Phyml as `Anomura_ind_final.phyml`. There is no need to save your temporary file.

> **Warning:** If your temporary combined matrices contain more than 10 taxa do not use the ienum in TNT to calculate the mini-supertree, use a heuristic method.

We have introduced another *MRP_Outgroup*, so this needs deleting (see above). Name your new file `Anomura_ind_final2.phyml`

5.8 Check data

This stage makes sure that the data is suitable for inclusion in the final supertree analysis. The first step is to create a data summary. This creates a list of useful information, such as taxa and characters. The information is printed alphabetically, which makes it easy to check for final errors. Although this is not necessary, it allows manual checking of the data, e.g. were genera replaced where species are also in the dataset?

Have a look in the file output and check that everything looks correct. If not, go back and fix things. Note that some of the statistics in the file might be useful - how many trees, over what years the data are from, types of characters in the dataset, etc.

The final step is to ensure that there is sufficient taxonomic overlap between source trees. We need to check that all the trees share at least two taxa with another source tree. You may also want to experiment with using higher numbers. The output can either be a simple yes/no or graphical output. Graphical output can either be a detailed view where a graph is produced whereby each source is a vertex and edges are drawn between sources that share the required number of taxa (Fig 5.8) . In this view *all* nodes should be blue, with no red (unconnected) nodes. However, for large datasets, this consumes a lot of memory and can take a long time to calculate. Instead use the normal view where connected trees compose a node in the graph (Fig 5.7). In this view there should be a single node only.

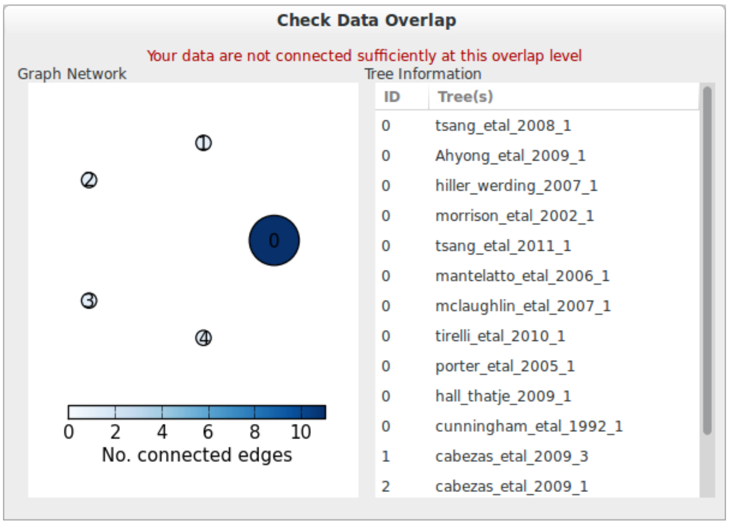

Figure 5.7: Graphical view of data overlap. For a correctly connected dataset there should be no unconnected nodes – i.e. there should be a single node. These data are not sufficiently well connected.

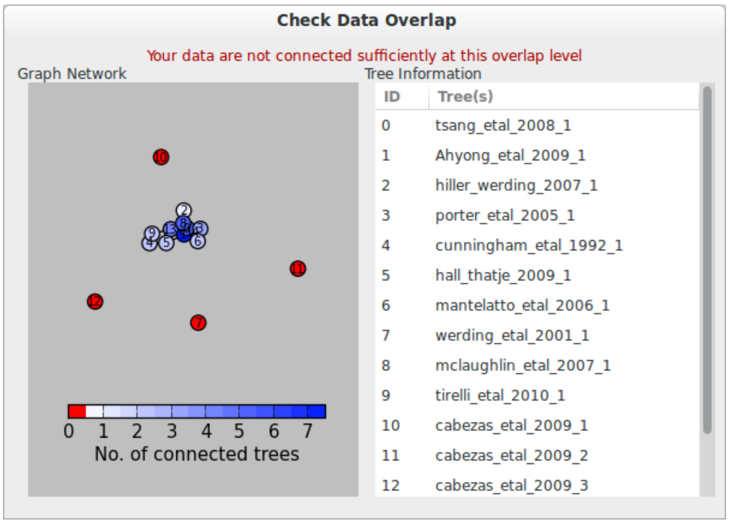

Figure 5.8: Detailed graphical view of data overlap. There should be no red nodes in a dataset that is well connected.

To carry out this step on our data in the CLI run this command:

stk data_overlap Anomura_ind_final2.phyml

It will return a message saying your data are not sufficiently well connected. We can find out which trees are not connected using:

stk data_overlap -g overlap_2.png -d Anomura_ind_final.phyml

Using the GUI, use *STK Functions* → *Check data overlap*. Click *Check overlap* and it will return a message about insufficient overlap. Run it again, with graphical output and you will see the following output.

Remove the following sources from the dataset (the sources contain all the trees that do not contain sufficient overlap):

- Cabezas et al 2009
- Werding et al 2001

You should then have 12 trees remaining. Remove the above and regenerate the overlap graphic – this time it should return a message saying your data are sufficiently well connected. Save your data to `Anomura_final.phyml`.

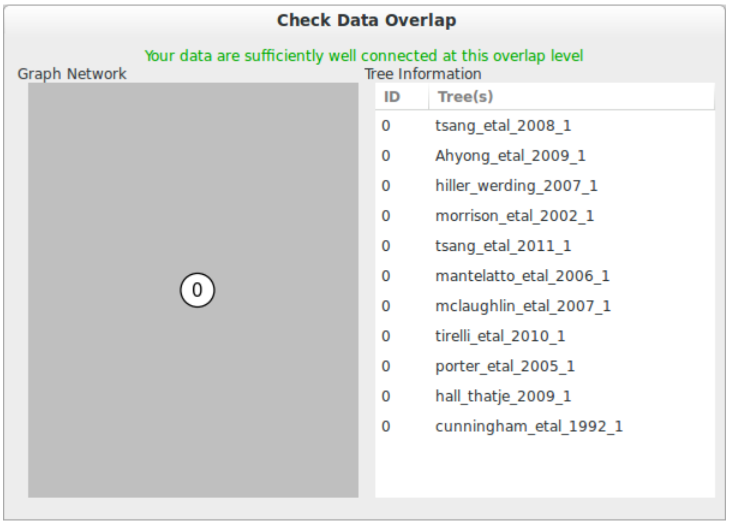

Figure 5.9: Graphical view of data overlap. For a correctly connected dataset there should be no unconnected nodes – i.e. there should be a single node. These data are now well connected.

5.9 Create matrix or export final tree set

You now have a dataset ready for creating a supertree. If you are using an algorithm that requires a matrix representation then the final step is to create a matrix.

Open `Anomura_final.phyml` and use *STK Functions* → *Create matrix* and fill in the GUI to create a matrix. Create a TNT matrix and save to `Anomura_matrix.tnt`

Alternatively, use:

stk create_matrix Anomura_final.phyml Anomura_matrix.tnt

The `Anomura_final.phyml` is included in the tutorial for comparison to yours.

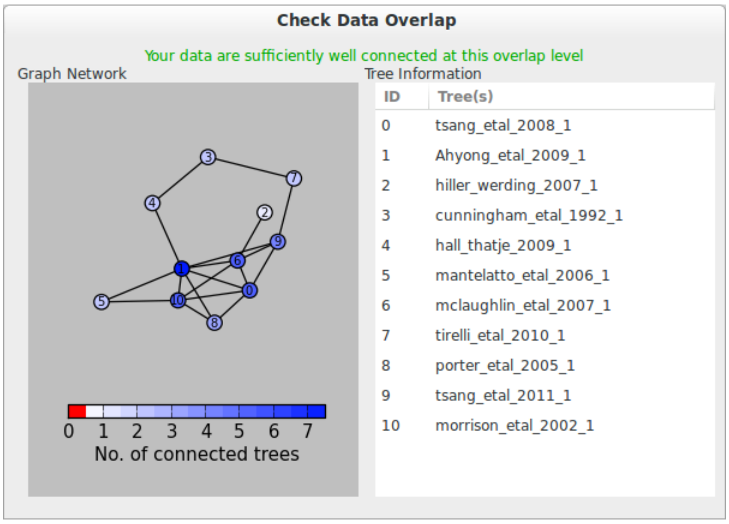

Figure 5.10: Detailed graphical view of data overlap. There are now no red nodes.

You can then load this matrix into TNT and generate your supertree using any suitable method. You can of course change the output format suitable for PAUP* or any other tree building software.

If you are using an algorithm that requires a set of trees then you can export your trees using *File* → *Export trees*.

CHAPTER

SIX

STK API

The STK has been split such that the main functionality can be imported into your own Python script and used programmatically. If you have downloaded the source package there are a number of example scripts in the stk/scripts directory. If you know Python, simply import the stk.supertree_toolkit module. If you don't, then go and learn Python – it's very useful.

Below is a description of the functions that are available in the API.

`stk.supertree_toolkit.`**`add_historical_event`**(*XML*, *event_description*)
Add a historial_event element to the XML. The element contains a description of the event and the the current date will ba added

`stk.supertree_toolkit.`**`all_sourcenames`**(*XML*)
Create a sensible sourcename for all sources in the current dataset. This includes appending a, b, etc for duplicate names.

`stk.supertree_toolkit.`**`amalgamate_trees`**(*XML*, *format='nexus'*, *anonymous=False*, *ignoreWarnings=False*)
Create a string containing all trees in the XML. String can be formatted to one of Nexus, Newick or TNT. Only Nexus formatting takes into account the anonymous flag - the other two are anonymous anyway Any errors and None is returned - check for this as this is the callers responsibility

`stk.supertree_toolkit.`**`check_subs`**(*XML*, *new_taxa*)
Check a subs file and issue a warning if any of the incoming taxa are not already in the dataset. This is often what is wanted, but sometimes it is not. We run this before we do the subs to alert the user of this but they may continue

`stk.supertree_toolkit.`**`clean_data`**(*XML*)
Cleans up (i.e. deletes) non-informative trees and empty sources Same function as check data, but instead of raising message, simply fixes the problems.

`stk.supertree_toolkit.`**`create_matrix`**(*XML*, *format='hennig'*, *quote=False*, *taxonomy=None*, *outgroups=False*, *ignoreWarnings=False*)
From all trees in the XML, create a matrix

`stk.supertree_toolkit.`**`create_matrix_from_trees`**(*trees*, *format='hennig'*)
Given a dictionary of trees, create a matrix

`stk.supertree_toolkit.`**`create_name`**(*authors*, *year*, *append=''*)
Construct a sensible from a list of authors and a year for a source name. Input: authors - list of last (family, sur) names (string).

year - the year (string). append - append something onto the end of the name.

Output: source_name - (string)

`stk.supertree_toolkit.`**`create_subset`**(*XML*, *search_terms*, *andSearch=True*, *includeMultiple=True*, *ignoreWarnings=False*)

Create a new dataset which is a subset of the incoming one. searchTerms is a dict, with the following keys: years - list consisting of the years to include. An entry can contain two years seperated by -. A range will then

be used.

characters - list of charcters to include character_types - list of character types to include (Molecular, Morphological, Behavioural or Other) analyses - list of analyses to include (MRP, etc) taxa - list of taxa that must be in a source tree fossil - all_fossil or all_extant

Multiple requests produce *and* matches (so between 2000-2010 *and* Molecular *and* contain Gallus gallus) unless andSearch is false. If it is, an *or* search is used. So the example would be years 2000-2010 *or* Molecular *or* contain Gallus gallus

includeMultiple means that a source can contain Molecular and Morophological characters and match Molecular (or, indeed, Morpholoigcal). Set to False to include if it's *only* Molecular you're after (i.e. trees with mixed character sets will be ignored). This applies to characters and character_types only (as the other terms don't make sense with this off).

Note: this funtion is not (yet) taxonomically aware, so Galliformes will only return trees that actually have a leaf called Galliformes. Gallus gallus will not match.

Also note: The tree strings are searched for taxa, not the taxa elements (which are optional)

A new PHYML file will be produced. The calling function must do something sensible with that

`stk.supertree_toolkit.`**`data_independence`**(*XML*, *make_new_xml=False*, *ignoreWarnings=False*)

Return a list of sources that are not independent. This is decided on the source data and the characters.

`stk.supertree_toolkit.`**`data_overlap`**(*XML*, *overlap_amount=2*, *filename=None*, *detailed=False*, *show=False*, *verbose=False*, *ignoreWarnings=False*)

Calculate the amount of taxonomic overlap between source trees. The output is a True/False by default, but you can specify an optional filename, which will save a nice graphic. For the GUI, the output can also be a PNG graphic to display (and then save).

If filename is None, no graphic is generated. Otherwise a simple graphic is generated showing the number of cluster. If detailed is set to true, a graphic is generated showing *all* trees. For data containing >200 source tres this could be very big and take along time. More likely, you'll run out of memory.

`stk.supertree_toolkit.`**`data_summary`**(*XML*, *detailed=False*, *ignoreWarnings=False*)

Creates a text string that summarises the current data set via a number of statistics such as the number of character types, distribution of years of publication, etc.

Up to the calling function to display string nicely

`stk.supertree_toolkit.`**`export_bibliography`**(*XML*, *filename*, *format='bibtex'*)

Export all source papers as a bibliography in either bibtex, xml, html, short or long formats

`stk.supertree_toolkit.`**`get_all_characters`**(*XML*, *ignoreErrors=False*)

Returns a dictionary containing a list of characters within each character type

`stk.supertree_toolkit.`**`get_all_source_names`**(*XML*)
From a full XML-PHYML string, extract all source names.

`stk.supertree_toolkit.`**`get_all_taxa`**(*XML*, *pretty=False*, *ignoreErrors=False*)
Produce a taxa list by scanning all trees within a PHYML file.

The list is return sorted (alphabetically).

Setting pretty=True means all underscores will be replaced by spaces

`stk.supertree_toolkit.`**`get_analyses_used`**(*XML*, *ignoreErrors=False*)
Return a sorted, unique array of all analyses types used in this dataset

`stk.supertree_toolkit.`**`get_character_numbers`**(*XML*, *ignoreErrors=False*)
Return the number of trees that use each character

`stk.supertree_toolkit.`**`get_character_types_from_tree`**(*XML*, *name*, *sort=False*)
Get the character types that were used in a particular tree

`stk.supertree_toolkit.`**`get_characters_from_tree`**(*XML*, *name*, *sort=False*)
Get the characters that were used in a particular tree

`stk.supertree_toolkit.`**`get_characters_used`**(*XML*)
Return a sorted, unique array of all character names used in this dataset

`stk.supertree_toolkit.`**`get_fossil_taxa`**(*XML*)
Return a list of fossil taxa

`stk.supertree_toolkit.`**`get_outgroup`**(*XML*)
For each tree, get the outgroup defined in the schema

`stk.supertree_toolkit.`**`get_publication_year_tree`**(*XML*, *name*)
Return a dictionary of years and the number of publications within that year

`stk.supertree_toolkit.`**`get_publication_years`**(*XML*)
Return a dictionary of years and the number of publications within that year

`stk.supertree_toolkit.`**`get_taxa_from_tree`**(*XML*, *tree_name*, *sort=False*)
Return taxa from a single tree based on name

`stk.supertree_toolkit.`**`get_weights`**(*XML*)
Get weights for each tree. Returns dictionary of tree name (key) and weights (value)

`stk.supertree_toolkit.`**`import_bibliography`**(*XML*, *bibfile*, *skip=False*)
Create a bunch of sources from a bibtex file. This includes setting the sourcenames for each source.

`stk.supertree_toolkit.`**`import_tree`**(*filename*, *gui=False*, *tree_no=-1*)
Takes a NEXUS formatted file and returns a list containing the tree strings

`stk.supertree_toolkit.`**`import_trees`**(*filename*)
Return an array of all trees in a file. All formats are supported that we've come across but submit a bug if a (common-ish) tree file shows up that can't be parsed.

`stk.supertree_toolkit.`**`load_phyml`**(*filename*)
Super simple function that returns XML string from PHYML file

`stk.supertree_toolkit.`**`obtain_trees`**(*XML*)
Parse the XML and obtain all tree strings Output: dictionary of tree strings, with key indicating treename (unique)

stk.supertree_toolkit.**parse_subs_file**(*filename*)
Reads in a subs file and returns two arrays: new_taxa and the corresponding old_taxa

None is used to indicated deleted taxa

stk.supertree_toolkit.**permute_tree**(*tree*, *matrix='hennig'*, *treefile=None*, *verbose=False*)
Permute a tree where there is uncertianty in taxa location. Output either a tree file or matrix file of all possible permutations.

Note this is a recursive algorithm.

stk.supertree_toolkit.**read_matrix**(*filename*)
Read a Nexus or Hennig formatted matrix file. Returns the matrix and taxa.

stk.supertree_toolkit.**replace_genera**(*XML*, *dry_run=False*, *ignoreWarnings=False*)
Remove all generic taxa by replacing them with a polytomy of all species in the dataset belonging to that genera

stk.supertree_toolkit.**safe_taxonomic_reduction**(*XML*, *matrix=None*, *taxa=None*, *verbose=False*, *queue=None*, *ignoreWarnings=False*)
Perform STR on data to remove taxa that provide no useful additional information. Based on PerEQ (Jeffery and Wilkson, unpublished).

stk.supertree_toolkit.**set_unique_names**(*XML*)
Ensures all sources have unique names.

stk.supertree_toolkit.**single_sourcename**(*XML*, *append=''*)
Create a sensible source name based on the bibliographic data. XML should contain the xml_root for the source that is to be altered only. NOTE: It is the responsibility of the calling process of this

function to check for name uniqueness.

stk.supertree_toolkit.**subs_file_from_str**(*str_output*)
From the textual output from STR (safe_taxonomic_reduction), create the subs file to put the C category taxa back into the dataset. We work with the text out as it's the same as PerlEQ, which means this might work from them also...

stk.supertree_toolkit.**subs_from_csv**(*filename*)
Create taxonomic subs from a CSV file, where the first column is the old taxon and all other columns are the new taxa to be subbed in-place

stk.supertree_toolkit.**substitute_taxa**(*XML*, *old_taxa*, *new_taxa=None*, *only_existing=False*, *ignoreWarnings=False*, *verbose=False*, *skip_existing=False*)
Swap the taxa in the old_taxa array for the ones in the new_taxa array

If the new_taxa array is missing, simply delete the old_taxa

only_existing will ensure that the new_taxa are already in the dataset

Returns a new XML with the taxa swapped from each tree and any taxon elements for those taxa removed. It's up to the calling function to do something sensible with this infomation

stk.supertree_toolkit.**substitute_taxa_in_trees**(*trees*, *old_taxa*, *new_taxa=None*, *only_existing=False*, *ignoreWarnings=False*, *verbose=False*)

Swap the taxa in the old_taxa array for the ones in the new_taxa array

If the new_taxa array is missing, simply delete the old_taxa

only_existing will ensure only taxa in the dataset are subbed in.

Returns a new list of trees with the taxa swapped from each tree It's up to the calling function to do something sensible with this infomation

PYTHON MODULE INDEX

S

stk.supertree_toolkit, 37

INDEX

A

add_historical_event() (in module stk.supertree_toolkit), 37
all_sourcenames() (in module stk.supertree_toolkit), 37
amalgamate_trees() (in module stk.supertree_toolkit), 37

B

bibtex, 9

C

check_subs() (in module stk.supertree_toolkit), 37
clean_data, 21
clean_data() (in module stk.supertree_toolkit), 37
CLI, 18
convert_files, 22
copy, 10
create_matrix, 21
create_matrix() (in module stk.supertree_toolkit), 37
create_matrix_from_trees() (in module stk.supertree_toolkit), 37
create_name() (in module stk.supertree_toolkit), 37
create_subset, 21
create_subset() (in module stk.supertree_toolkit), 38

D

data summary, 10
data_ind, 21
data_independence() (in module stk.supertree_toolkit), 38
data_overlap, 21
data_overlap() (in module stk.supertree_toolkit), 38
data_summary, 21
data_summary() (in module stk.supertree_toolkit), 38

E

export_bib, 20
export_bibliography() (in module stk.supertree_toolkit), 38
export_data, 20
export_trees, 20

G

get_all_characters() (in module stk.supertree_toolkit), 38
get_all_source_names() (in module stk.supertree_toolkit), 39
get_all_taxa() (in module stk.supertree_toolkit), 39
get_analyses_used() (in module stk.supertree_toolkit), 39
get_character_numbers() (in module stk.supertree_toolkit), 39
get_character_types_from_tree() (in module stk.supertree_toolkit), 39
get_characters_from_tree() (in module stk.supertree_toolkit), 39
get_characters_used() (in module stk.supertree_toolkit), 39
get_fossil_taxa() (in module stk.supertree_toolkit), 39
get_outgroup() (in module stk.supertree_toolkit), 39
get_publication_year_tree() (in module stk.supertree_toolkit), 39
get_publication_years() (in module stk.supertree_toolkit), 39
get_taxa_from_tree() (in module stk.supertree_toolkit), 39
get_weights() (in module stk.supertree_toolkit), 39
grouping, 10

I

import_bibliography() (in module stk.supertree_toolkit), 39
import_data, 20
import_tree() (in module stk.supertree_toolkit), 39

import_trees() (in module stk.supertree_toolkit), 39

L

load_phyml() (in module stk.supertree_toolkit), 39

O

obtain_trees() (in module stk.supertree_toolkit), 39

P

parse_subs_file() (in module stk.supertree_toolkit), 39
paste, 10
permute_tree() (in module stk.supertree_toolkit), 40
permute_trees, 21

R

read_matrix() (in module stk.supertree_toolkit), 40
replace_genera, 21
replace_genera() (in module stk.supertree_toolkit), 40

S

safe_taxonomic_reduction, 21
safe_taxonomic_reduction() (in module stk.supertree_toolkit), 40
set_unique_names() (in module stk.supertree_toolkit), 40
single_sourcename() (in module stk.supertree_toolkit), 40
slicing, 10
stk.supertree_toolkit (module), 37
sub_taxa, 21
subs_file_from_str() (in module stk.supertree_toolkit), 40
subs_from_csv() (in module stk.supertree_toolkit), 40
substitute_taxa() (in module stk.supertree_toolkit), 40
substitute_taxa_in_trees() (in module stk.supertree_toolkit), 40
